# Integrated assessment of medicinal rhubarb by combination of delayed luminescence and HPLC fingerprint with emphasized on bioactivities based quality control

**DOI:** 10.1186/s13020-020-00352-8

**Published:** 2020-07-14

**Authors:** Mengmeng Sun, Hongwei Wu, Min He, Yusheng Jia, Lixue Wang, Ting Liu, Lianqiang Hui, Li Li, Shengli Wei, Eduard Van Wijk, Roeland Van Wijk, Karl Wah-Keung Tsim, Chun Li, Mei Wang

**Affiliations:** 1 LU-European Center for Chinese Medicine and Natural compounds, Institute of Biology, Leiden University, Sylviusweg 72, 2333 BE Leiden, The Netherlands; 2 Institute of Chinese Materia Medica, China Academy of Chinese Medical Sciences, Beijing, 100700 China; 3 Changchun University of Chinese Medicine, No. 1035, Boshuo Rd, Jingyue Economic Development District, Changchun, 130117 China; 4 SU BioMedicine, Post Bus 546, 2300 AM Leiden, The Netherlands; 5 Shenzhen HUAKAI TCM and Natural Medicine Research Center, NO. 2, Boya Building, Zone A, Dawang Cultural and Creative Industrial Park, Wutong Mountain, No. 197, Kengbei Village, Luohu District, Shenzhen, 518114 China; 6 Capital Medical University subsidiary Beijing Hospital of Traditional Chinese Medicine, No. 23 Backstreet of Art Gallery, Dongcheng District, Beijing, 100010 China; 7 Beijing Institute of Chinese Medicine, No. 13 Shuiche Alley Xinjiekou, Xicheng District, Beijing, 100035 China; 8 School of Chinese Pharmacy, Beijing University of Chinese Medicine, No. 6 Wangjing Zhonghuan South Street, Chaoyang District, Beijing, 100102 China; 9 Meluna Research, Koppelsedijk 1-a, 4191LC, Geldermalsen, The Netherlands; 10 Division of Life Science and Center for Chinese Medicine R&D,, Kowloon, Hong Kong, China, The Hong Kong University of Science and Technology, Kowloon, Hong Kong China; 11 SKL of Quality Research in Chinese Medicine, Institute of Chinese Medical Sciences, University of Macau, N22 Avenida da Universidade, Taipa, Macau, China

**Keywords:** Rhubarb, Fingerprint, Delayed luminescence, Cathartic activity, Quality control

## Abstract

**Background:**

To promote herbal medicine depends largely on its quality. Chromatographic fingerprint is a frequent approach for quality assessment of herbs however with challenges on robust and reproducibility. To develop rapid, cheap and comprehensive measurements as complementary tools for herbal quality control are still urgently needed. Moreover, biological activities are essential for herbal quality, and should be taken into consideration with emphasized in quality control.

**Methods:**

In this research, HPLC fingerprint and delayed luminescence (DL, a rapid and systematic tool) were used to measure the rhubarb samples of multiple species. Statistics were explored to classify these rhubarb samples using data obtained from two analytic methods. In addition, DL properties were linked to specific chemical components which may reflect bioactivities of rhubarb using Spearman’s rank correlation. Moreover, mice model was used to evaluate the cathartic effect between rhubarb samples stratifying by two analytic methods.

**Results:**

We found that there was no significant difference of chemical fingerprints and DL signals among the different species of medicinal rhubarb. However, our results show a high similarity between HPLC fingerprint analysis and DL measurements in classification of these rhubarb samples into two sub-groups. In addition, the two sub-groups of rhubarb samples that may have different cathartic activities.

**Conclusion:**

This approach provides new leads for development of herbal quality assessment based on bioactivity. In conclusion, integrated assessment by measuring HPLC fingerprint and DL with emphasized on bioactivity may provide novel strategy for herbal quality control.

## Background

China has long history for using of Chinese herbal medicine for maintaining of health and treating of disease and an increasingly global level during the last decades is taken place [1, 2]. Recently, World Health Organization recognizes traditional Chinese medicine in its influential global medical compendium, which shows a significant acceptance worldwide [3]. However, to further promote Chinese herbal medicine depends largely on its quality control which is directly reflected to its safety and efficacy [4]. Bio-active constituents of herbs are usually very complex, multiple constituents are often synergistically responsible for their multi-target effects [5]. There has been an important shift from evaluating individual compounds to evaluating profiling of multiple-constituent chemical components in herbal quality control [6]. Generally, the chromatographic fingerprint is considered to be one of the most important approaches for quality assessment of herbs. Since chromatographic fingerprint pattern can reflect systematically the multi-chemical components and/or chemically characteristics in specific herbs [5]. Chromatographic fingerprint is usually used to quantify certain marker compounds and to evaluate similarity, authenticity and stability of herbal materials etc. [7]. But it still has some limitations caused by the sensitivity of instrument, robust and reproducibility of methodology [8]. This means that relatively long period of time for sample preparation and analysis, as well as relatively high testing costs are the bottle neck of this technology [9]. Therefore, to develop direct, rapid, cheap and comprehensive methods as complementary tools for herbal quality assessment are still urgently needed. In addition, linking the bioactivities with the phytochemical constituents of herbs is the most important challenges for quality control. Bioactivity (safety and efficacy) based herbal quality research, such as dosage dependent efficiency and toxic effects relationship with chemical components, have to be further developed [10]. Therefore, an integrated research strategy which combines Chemical fingerprint pattern, rapid detection technology and herbal bioactivity assay may reveal novel insights in comprehensive quality control of herbs.

Nuclear magnetic resonance (NMR), Raman and infrared spectroscopy are usually combined with chromatographic fingerprint in herbal quality control [11, 12]. Recently, delayed luminescence (DL) has been used to study the features of dry powders prepared from Chinese herbal materials [13–17]. DL is the long-term decay of weak photon emissions from various materials (e.g., cell, organism, food, seed, plants and heavy metal contaminate etc.) following exposure to excitation light with a wavelength of 400–800 nm [18–21]. DL provides a new method for measuring herbal materials which can be recognized as a rapid, direct and sensitive indicator of a wide range of herbs prepared in different conditions including the grown environments, the age, the processing statues as well as the therapeutic properties of herbs [14–16]. Thus, DL becomes a promising technique for herbal quality control, and it may become a novel tool to combine with other analytic technological platform.

**Fig. 1:**
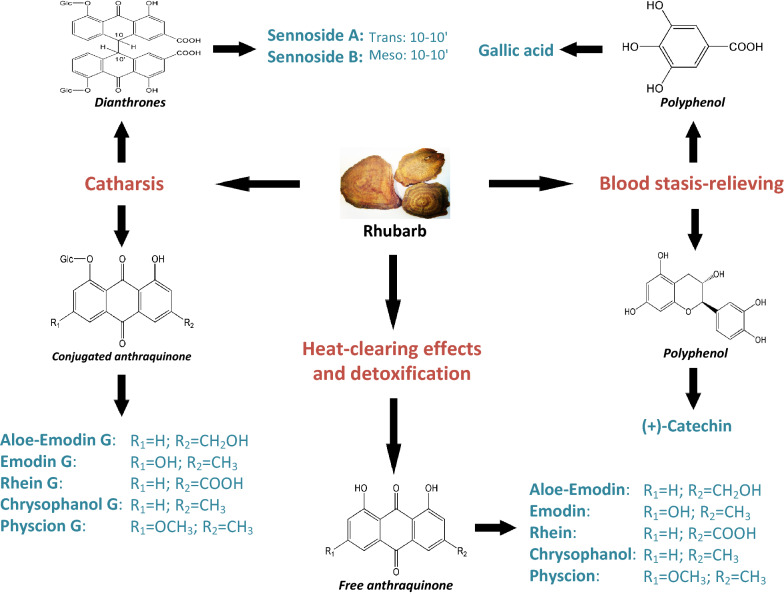
Schematic diagram of the chemical components in rhubarb and their role in Chinese herbal medicine-based concepts. Different chemical compounds in rhubarb correspond to various therapeutic properties. The terms in italics under the chemical structure indicate the structural characteristics of the chemical components. “Glc” in the chemical structure indicates a glycoside. “G” in the names of chemical constituents indicate glycosides. Conjugated anthraquinone represents anthraquinone glycosides with O-glycosides, where the aglycone moiety is an 8-dihydroxyanthraquinone derivative. Additional structures of conjugated anthraquinones not shown here are published elsewhere [24]

Rhei Rhizoma (i.e., rhubarb) is a famous Chinese herbal drug. The dried roots and rhizomes of *Rheum palmatum* L., *Rheum tanguticum* Maxim. ex Balf., and *Rheum officinale* Bail. are officially included in various editions of the Chinese Pharmacopoeia [22]. Rhubarb is a traditional herbal remedy with many therapeutic properties, including heat-clearing and detoxification effects, catharsis and removal of blood stasis according to Chinese medicine practice [22]. Recently, chemical and pharmacological studies have shown that the bioactivities of secondary metabolites in rhubarb—including anthraquinone derivatives and polyphenol constituents—correspond with different traditional therapeutic properties (Fig. 1). For instance, rhubarb’s detoxification property is reflected largely by its antibacterial activity, which is related primarily to free anthraquinones such as aloe-emodin, emodin and rhein [23]. Rhubarb’s cathartic property has been mainly attributed to the presence of glycoside-containing component such as anthraquinone glycosides and sennosides [24, 25]. Rhubarb’s blood stasis–relieving property is due to polyphenol compounds, for example, (+)-catechin and gallic acid [26, 27]. Given its various bioactive components and therapeutic properties, rhubarb has been used to study its high performance liquid chromatography (HPLC) fingerprint characteristics and DL properties, respectively [14, 28]. In additional, previous study showed that both HPLC analysis and DL measurements revealed that the bioactive composition of samples from one specific rhubarb specie (*Rheum palmatum* L.) are affected by environmental factor [14]. However, the integrated assessment of rhubarb materials with multiple medical species, which officially included in Chinese Pharmacopoeia, by combination of chemical fingerprint, DL and bioactivities is still limited.

In order to evaluate whether DL properties can be used to create a similar quality assessment of rhubarb materials with multiple species compared to HPLC fingerprint analysis. We performed both HPLC fingerprint analysis and DL measurements in the same rhubarb materials. In addition, animal model was used to validate the cathartic activity in two sub-groups of rhubarb materials discriminated by both HPLC fingerprint analysis and DL measurements. Our results show a high similarity between HPLC fingerprint analysis and DL measurements in identification of rhubarb materials. In addition, the linking certain chemical components with bioactivities in animal model demonstrated a potential novel tool for comprehensive quality control in herbal medicines.

## Materials and methods

### Rhubarb materials and chemicals

28 batches of commercial rhubarb samples were purchased in herbal medicine markets located in different places of China (Table 1). 118 batches of wild rhubarb samples (55 batches of *Rheum palmatum* L. and 63 batches of *Rheum tanguticum* Maxim. ex Balf.) were obtained as gifted samples from Beijing Institute of Chinese Medicine. All rhubarb samples were verified by Prof. Hongwei Wu and deposited at China Academy of Chinese Medical Sciences, Beijing, China.

**Table 1:** Summary of the 28 commercial rhubarb samples

ID	Species	Batch	Place of purchase
S1	*Rheum officinale* Bail.	20,170,912	Enshi, Hubei
S2	*Rheum palmatum* L.	20,170,914	Bozhou, Anhui
S3	*Rheum tanguticum* Maxim. ex Balf.	20,170,930	Anguo, Hebei
S4	*Rheum tanguticum* Maxim. ex Balf.	20,170,914	Bozhou, Anhui
S5	*Rheum officinale* Bail.	20,170,930	Anguo, Hebei
S6	*Rheum palmatum* L.	20,170,914	Tanchang, Gansu
S7	*Rheum palmatum* L.	20,170,930	Anguo, Hebei
S8	*Rheum officinale* Bail.	20,170,512	Bozhou, Anhui
S9	*Rheum tanguticum* Maxim. ex Balf.	20,170,914	Bozhou, Anhui
S10	*Rheum palmatum* L.	20,170,914	Tanchang, Gansu
S11	*Rheum officinale* Bail.	20,171,122	Anguo, Hebei
S12	*Rheum tanguticum* Maxim. ex Balf.	20,171,122	Anguo, Hebei
S13	*Rheum officinale* Bail.	20,171,122	Bozhou, Anhui
S14	*Rheum officinale* Bail.	20,171,124	Bozhou, Anhui
S15	*Rheum tanguticum* Maxim. ex Balf.	20,171,124	Chengdu, Sichuan
S16	*Rheum officinale* Bail.	20,171,124	Anguo, Hebei
S17	*Rheum officinale* Bail.	20,171,125	Bozhou, Anhui
S18	*Rheum palmatum* L.	20,171,125	Tanchang, Gansu
S19	*Rheum palmatum* L.	20,171,127	Bozhou, Anhui
S20	*Rheum tanguticum* Maxim. ex Balf.	20,171,128	Bozhou, Anhui
S21	*Rheum officinale* Bail.	20,171,129	Bozhou, Anhui
S22	*Rheum tanguticum* Maxim. ex Balf.	20,180,323	Bozhou, Anhui
S23	*Rheum palmatum* L.	20,180,323	Bozhou, Anhui
S24	*Rheum officinale* Bail.	20,180,323	Bozhou, Anhui
S25	*Rheum officinale* Bail.	2,018,032	Bozhou, Anhui
S26	*Rheum officinale* Bail.	20,170,103	Chengdu, Sichuan
S27	*Rheum officinale* Bail.	Y20100118	Anguo, Hebei
S28	*Rheum officinale* Bail.	20,180,426	Chengdu, Sichuan

Gallic acid, aloe-emodin-8-O-beta-d-glucoside, sennoside A and chrysophanol-8-O-beta-d-glucoside were purchased from Push bio-thecnology Co., Ltd. (Chengdu, China). Emodin-1-O-beta-d-glucoside, (+)-catechin, emodin and chrysophanol were purchased from RCXD technological development Co., Ltd. (Beijing, China). Aloe-emodin, rhein and physcion were purchased from National Institutes for Food and Drug Control (Beijing, China). Physcion-8-O-beta-d-glucoside and emodin-8-O-beta-d-glucoside were purchased from Sichuan Xianxin Biotech Co., Ltd. (Chengdu, China). Chrysophanol-1-O-beta-d-glucoside was purchased from Chroma-Biotech Co., Ltd. (Chengdu, China). Rhein-8-O-beta-d-glucoside was purchased from Yuanye Bio-Technology Co., Ltd. (Shanghai, China). The purity of all reference compounds was > 98%.

### Chemical analyses

#### Sample preparation and HPLC analysis for commercial rhubarb materials

Powdered commercial rhubarb samples (1-g) were extracted with 50 ml 70% methanol (v/v) using a model KQ250DB ultrasonication device (200 W, 40 HZ; Kunshan Ultrasonic Instruments Co., Ltd., Kunshan City, China) for 1 h. The extracted solution was prepared by the method of weight relief, in which we compensated for any weight lost during the extraction procedure. The weight lost during the extraction procedure was replaced with 70% methanol (v/v). The solution was then filtered through a 0.45-μm membrane and analyzed by HPLC.

HPLC analysis was performed using a Shimadzu LC-2010AHT system (Shimadzu, Tokyo, Japan). Chromatographic analysis was conducted using a Welch Ultimate XB-C18 column (4.6 mm × 250 mm, particle size: 5 μm) maintained at 30 °C. The detection wavelength was 280 nm. The mobile phase consisted of methanol (A) and 0.1% (v/v) aqueous phosphoric acid (B) with a gradient program of 5–30% (A) at 0–20 min, 30–60% (A) at 20–75 min, 60–100% (A) at 75–105 min, 100–100% (A) at 105–110 min. The flow was 1 ml/min. The injected volume was 10 μl, and the standard solution containing 15 bioactive reference compounds was prepared in methanol. The method is well-established and validated, showing reasonable reproducibility and repeatability for each chemical constituent.

#### Sample preparation and HPLC analysis for wild rhubarb materials

In order to obtain the contents of individual compounds and total amounts of free anthraquinones, glycoside-containing compounds and polyphenol compounds in wild rhubarb samples, HPLC analysis was performed using an Agilent 1100 system (Agilent Technologies, Palo Alto, CA). The sample preparation, HPLC analysis methods and statistical methods have been reported previously [14]. The sum of individual compounds in the category of free anthraquinones, glycoside-containing component and polyphenol polyphenol component was used to perform further statistical analysis, respectively.

### DL measurements

#### Sample preparation

Both commercial and wild rhubarb samples were crushed using a model QE-100 grinder (Yili Company, Zhejiang Province, China), and 150-μm particles were selected using a standard sieve. Thereafter, the samples were stored in a dark box containing 3–5-mm silica gel (Boom BV, Meppel, the Netherlands) at room temperature for ≥ 16 h before DL measurements were performed [15].

#### DL measurement

DL was measured using a previously established protocol for herbal drugs [15]. The instrument for measuring DL (Meluna Research, the Netherlands) included a dark sample chamber with a vertically positioned photomultiplier tube (model 9558QB; Electron Tubes Enterprises Ltd., Ruislip, UK). The sample chamber was kept at 22 °C. The cathode end of the PMT has a diameter of 51 mm and is sensitive at 300–800 nm. The PMT was cooled to − 25 °C in order to reduce the dark count rate to 10 counts per second. The photon emission signal was amplified using fast preamplifier (model 9301; ORTEC, Oak Ridge, TN). A personal computer containing counting card (model 6602; National Instruments, Austin, TX) was used for signal data acquirement. Each batch of rhubarb powder was used to prepare 1-g sample. Each 1-g sample was placed in a Petri dish (diameter: 1 cm) and excited for 10 s using a white halogen source (model 284–2812; Philips, Germany). The DL of each rhubarb sample was measured three consecutive times. The total number obtained from all three measurements in each sample was used to analyze the DL properties of that particular rhubarb sample. DL kinetics were obtained by recording the number of counts in consecutive 0.05-s periods for a total of 30 s, resulting in a total of 600 data points.

### Cathartic activity tests in mice model

#### Animals

The male ICR mice (weighing 20–24 g, Specific Pathogen Free) were purchased from Beijing Vital River Laboratory Animal Technology Co., Ltd. (License number: SCXK 2016-0006). The mice were raised in the animal rooms of Institute of Chinese Materia Medica, China Academy of Chinese Medical Sciences. The animal rooms were exposed to artificial light for 12 h per day. The ambient temperature was maintained at 20–24 °C and the humidity was 40–70%. The animal rooms were ventilated 15 times per hour. The Administrative Panel on Laboratory Animal Care of Institute of Chinese Materia Medica, China Academy of Chinese Medical Sciences approved all experimental procedures (No. 20182020). All animal experiments were performed in accordance with institutional guidelines and ethics of China Academy of Chinese Medical Sciences and the National Institutes of Health guide for the care and use of Laboratory animals.

#### Rhubarb extract solution

Commercial rhubarb samples (S10 and S22) were extracted using reflux extraction with the 70% methanol, respectively. The obtained rhubarb extracts were re-dissolved using distilled water in order to obtain rhubarb extract solution. The rhubarb extract solution was given to mice by intragastrical gavage at three different doses. The administration of high dose group was equivalent to 5-g crude rhubarb/kg. The administration of medium dose group was equivalent to 2.5-g crude rhubarb/kg. The administration of low dose group was equivalent to 1-g crude rhubarb/kg.

#### Defecation test in mice

The 58 mice were randomly divided into seven treatment groups: control (8 mice), low dose of rhubarb S10 (8 mice), medium dose of rhubarb S10 (8 mice), high dose of rhubarb S10 (9 mice), low dose of rhubarb S22 (8 mice), medium dose of rhubarb S22 (8 mice) and high dose of rhubarb S22 (9 mice). All the mice were fasted for 4 h with free access to water prior to the experiments. After that, the mice in each treatment group were administered corresponding rhubarb extract solution. The normal control group was given an equal volume of normal saline. After thirty minutes of drug administration, each mouse was given 5% CMC-Na suspension containing 0.5% charcoal powder by intragastrical gavage. Thereafter, each mouse was immediately placed in a single cage, and the cleaning filter paper was located under the cage in order to record of defecation. The incubation period of charcoal powder-containing feces was recorded for each mouse, and the number of charcoal powder-containing feces and the total weight of feces were recorded after 5 h. The feces with stains on the filter paper were loose stools and those without stains were dry stools. The mice with loose stools were regarded as diarrhea. The number of mice with diarrhea in each group was recorded and the incidence of diarrhea was calculated. The incidence of diarrhea (%) = the number of mice with loose stools/total number of mice × 100.

#### Small intestine propelling test in mice

The 60 mice were randomly divided into seven treatment groups: control (8 mice), low dose of rhubarb S10 (8 mice), medium dose of rhubarb S10 (9 mice), high dose of rhubarb S10 (9 mice), low dose of rhubarb S22 (8 mice), medium dose of rhubarb S22 (9 mice) and high dose of rhubarb S22 (9 mice). All the mice were fasted for 24 h with free access to water prior to the experiments. After that, the mice in each treatment group were administered corresponding rhubarb extract solution. The normal control group was given an equal volume of normal saline. After 30 min of drug administration, each mouse was given 5% CMC-Na suspension containing 0.5% charcoal powder by intragastrical gavage. After 20 min of previous administration, the mice were executed by cervical dislocation. Thereafter, the small intestine from pylorus to the boundary of ileum and cecum was isolated. The small intestine is gently drawn into a straight line. The total length of the small intestine and the distance from pylorus to the front end of charcoal powder were measured in order to calculate the propelling ratio of charcoal powder. The charcoal powder propelling ratio (%) = the distance from pylorus to the front end of charcoal powder (cm)/total length of small intestine (cm) × 100 [29].

### Data processing and statistical analysis

#### Statistics of chemical data

The contents of fifteen compounds and the values of relative peak area of common peaks were analyzed using principal component analysis (PCA) and Orthogonal projections to latent structures discriminant analysis (OPLS-DA) in order to indicate the level of discrimination in those 28 batches of commercial rhubarb samples. The contents of obtained compounds were used to perform PCA analysis for the 118 batches of wild rhubarb samples. The PCA and OPLS-DA tools were provided in the MetaboAnalyst software package (http://www.metaboanalyst.ca). The total amounts of individual compounds in the category of free anthraquinones, glycoside-containing component and polyphenol polyphenol component of commercial and wild rhubarb samples, respectively, were calculated for next analysis. Subsequently, a two-tailed, unpaired Student’s *t* test was performed (SPSS version 23.0; IBM, Armonk, NY) to compare the different rhubarb sub-groups using the contents of identified individual compounds and chemical components; differences were considered significant at *p *< 0.05.

#### Statistics of DL data

The photon counts measured during the 30 s of each decay curve were used to calculate the properties of the following hyperbolic function [16]:

$$I_{\left( t \right)} = \frac{{I_{0} }}{{\left( {1 + \frac{t}{Tau}} \right)^{Beta} }}$$

$$T = \left( {e^{{\frac{1}{ Beta}}} - 1} \right) \times Tau$$

where Beta is an index factor associated with the rate of DL decay, I0 is the initial intensity of the DL curve, and T and Tau represents the decay time and DL characteristics, respectively. The properties of the three measurements were averaged and used to represent the DL properties of each batch of rhubarb. PCA, OPLS-DA and the hierarchical cluster analysis were used to indicate the level of discrimination between DL properties of 28 commercial rhubarb samples and 118 wild rhubarb samples, respectively, using tools provided in the MetaboAnalyst software package (http://www.metaboanalyst.ca). A two-tailed, unpaired Student’s t-test was used (SPSS version 23.0) to compare the DL properties between the rhubarb sub-groups identified from previous analyses; differences were considered significant at *p *< 0.05.

#### Correlation between identified compounds and DL properties

Spearman’s rank correlation (*ρ*) was used to quantify the correlation between the 15 identified compounds and DL properties in the commercial rhubarb samples. Linear relationship was defined as Spearman’s |*ρ*| > 0.30 [30, 31]. Thereafter, Cytoscape version 3.2.1 (www.cytoscape.org) was used to draw a network view, which was used visualize these correlations.

#### Statistics of animal study

The incubation period of charcoal powder-containing feces, the number of charcoal powder-containing feces, the total weight of feces, the incidence of diarrhea and the charcoal powder propelling ratio were analyzed using One-way analysis of variance (ANOVA) among different treatment groups (SPSS version 23.0). Least significant difference (LSD) analysis was used in the case of homogeneity of variance. Tamhane’s T2 test was used in the case of variance nonhomogeneity. The diarrhea ratio was analyzed by Crosstabs Chi square test, Fisher’s Exact Test was further used for inter-group comparisons if there were overall differences; differences were considered significant at *p *< 0.05.

## Results

### HPLC fingerprint analysis of the 28 commercial rhubarb samples

**Fig. 2:**
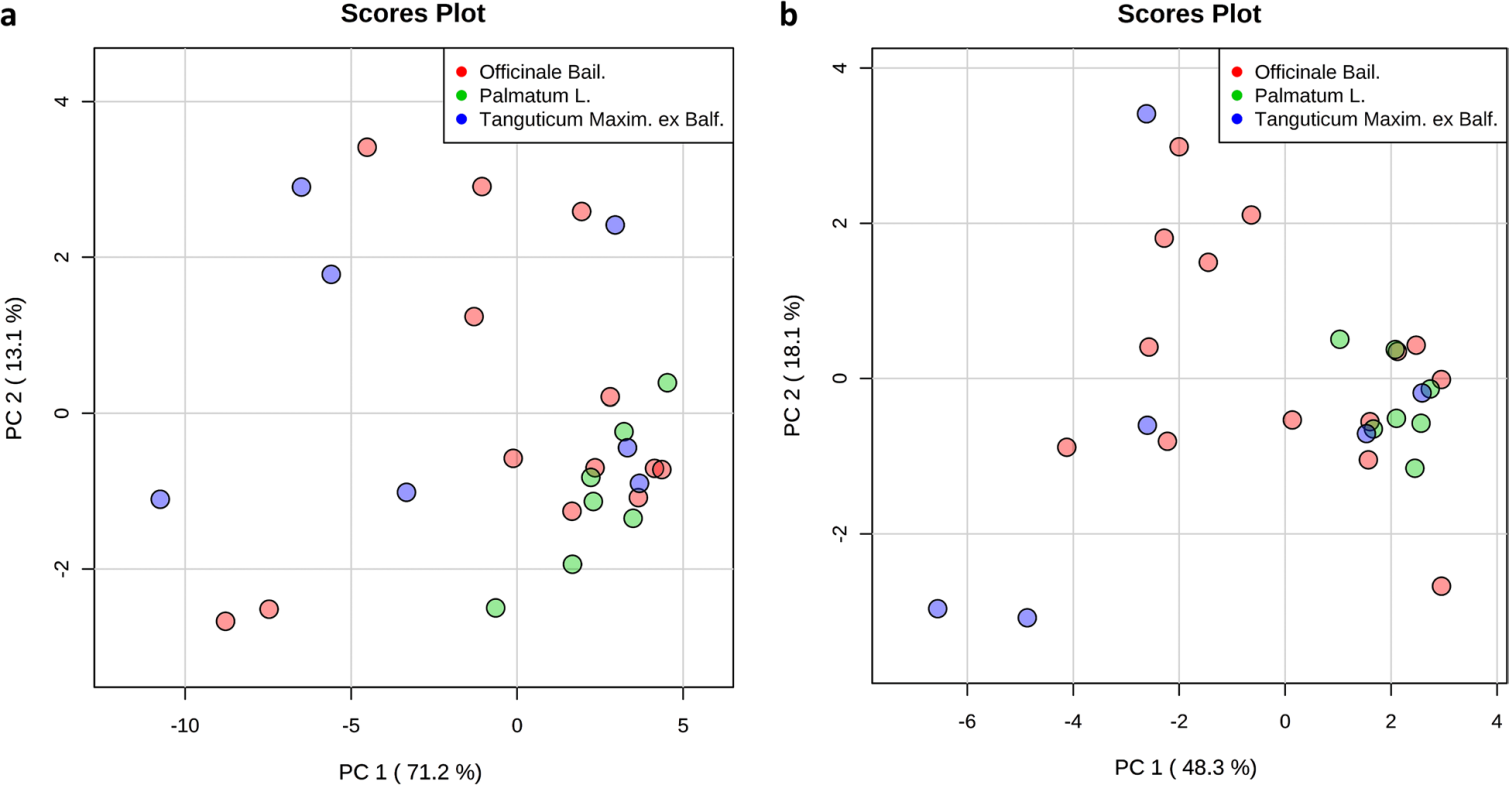
PCA scores obtained from the chemical data. **a** PCA score plots of the relative peak area obtained from all batches of commercial rhubarb samples; **b** PCA score plots of the contents of identified compounds obtained from all batches of commercial rhubarb samples

**Fig. 3:**
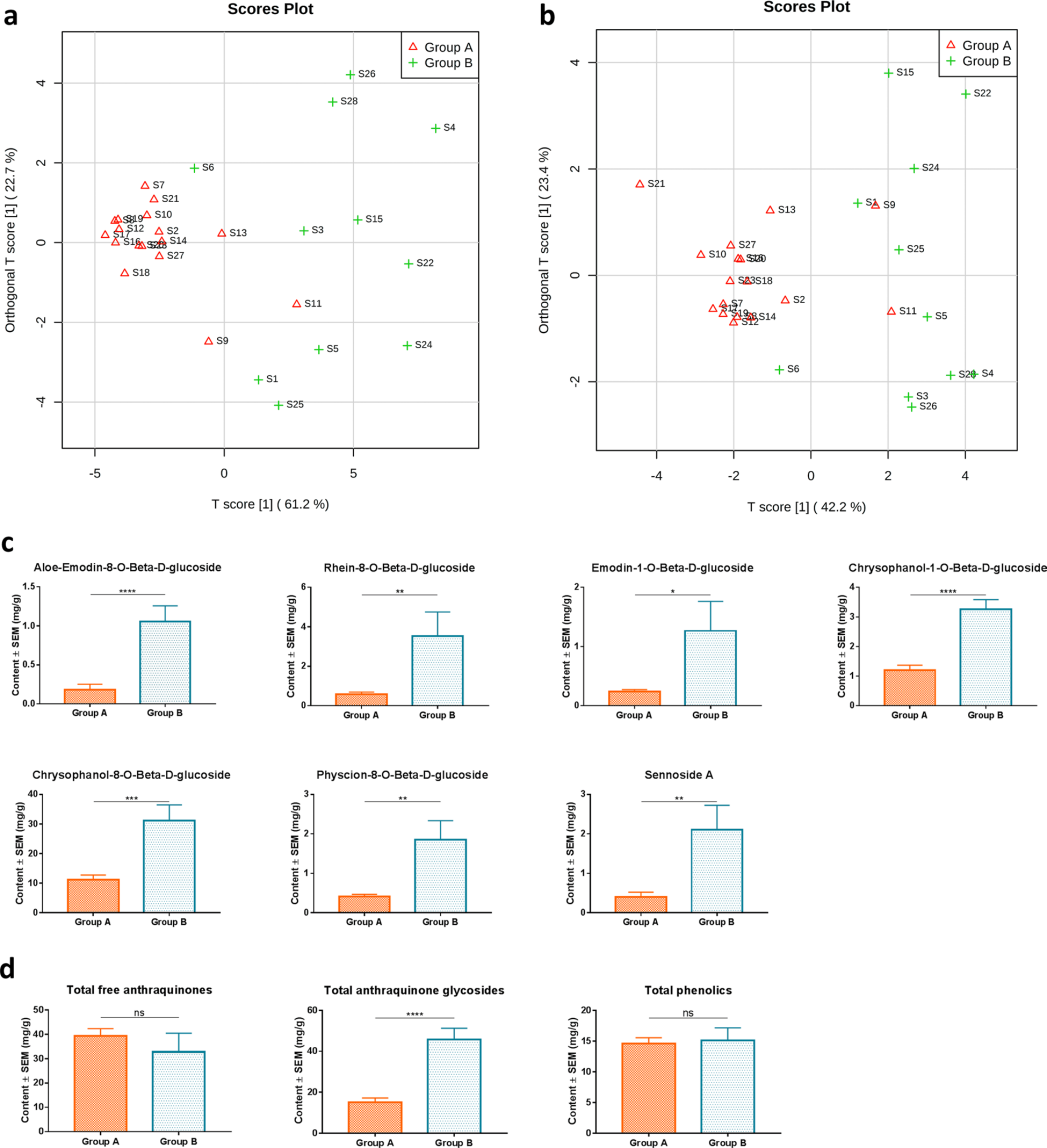
Chemical analysis of the commercial rhubarb samples. **a** OPLS-DA score plots of the relative peak area obtained from all batches of commercial rhubarb samples with cross-validation revealed predictive accuracy of 0.612 (Q^2^) and goodness-of-fit of 0.702 (R^2^), respectively; **b** OPLS-DA score plots of the contents of identified compounds obtained from all batches of commercial rhubarb samples with cross-validation revealed predictive accuracy of 0.541(Q^2^) and goodness-of-fit of 0.661 (R^2^), respectively; **c**, **d** Histograms comparing the contents of chemical compounds between two groups. *, *p* < 0.05; **, *p *< 0.01; ***, *p* < 0.001; ****, *p *< 0.0001; ns, no significant difference (two-tailed, unpaired Student’s t-test)

To evaluate the quality of rhubarb samples, HPLC fingerprint analysis was used to quantify the 28 batches of rhubarb samples. Twenty-eight common peaks were obtained in the fingerprint of those 28 rhubarb samples. In addition, fifteen compounds were identified by comparison with the chemical standards (Additional file 1: Fig. S1, S2; Table S1). Given the retention time of common peak No.13 was in the middle (Additional file 1: Fig. S1), and the separation and purity were better, common peak No. 13 was determined as reference peak. The ratios of the retention time and the peak area between all the common peaks and the common peak No. 13 were defined as the relative retention time and relative peak area (Additional file 1: Table S2, S3). In order to evaluate the similarity among the 28 commercial rhubarb samples, the obtained data was analyzed using the professional software named Similarity Evaluation System for HPLC (version 2004) [31]. Table 2 lists the similarity index, and the high similarity index represents the higher similarity of chemical profile between samples [32]. The results show that the similarities in rhubarb samples from the same species can have big variabilities. For example, the similarity between sample S3 and S4 was very low (Similarity index: 0.173). But the rhubarb samples from different species can have high similarity. For instance, the similarity index was 0.938 between rhubarb sample S2 and S8. This results may indicate that the different sub-species were not the major cause of chemical differences in these commercial rhubarb samples. To further evaluate the data, unsupervised PCA was applied to the chemical data for visualizing the variations among different rhubarb sub-species. The results illustrated that there was no significant difference between different sub-species of rhubarb samples (Fig. 2). Therefore, we mainly focused on the reference similarity index between the individual fingerprint and the standard fingerprint of the commercial rhubarb samples (Table 2). However, according to reference similarity index, we were able to see two sub-groups rhubarb samples. The samples with relatively high index were assigned to Group A, and the samples with relatively low index were assigned to Group B (Table 2). Next, a supervised clustering approach (OPLS-DA) was further performed to obtain the optimal separation using the chemical data. The results obtained from the OPLS-DA analysis revealed a reasonable separation between Group A and Group B (Fig. 3a, b). Next, the differences in the identified chemical compounds between the two sub-groups were analyzed using a two-tailed, unpaired Student’s t-test. The analysis revealed that the contents of seven compounds in group A were significantly lower than that in the group B, as well as the total content of glycoside-containing compounds (Fig. 3c, d).

**Table 2:** Similarity index of the 28 commercial rhubarb samples

	S1	S2	S3	S4	S5	S6	S7	S8	S9	S10	S11	S12	S13	S14	S15	S16	S17	S18	S19	S20	S21	S22	S23	S24	S25	S26	S27	S28
S1	1	0.673	0.614	0.105	0.916	0.548	0.6	0.611	0.843	0.651	0.837	0.669	0.661	0.686	0.359	0.662	0.653	0.713	0.635	0.688	0.682	0.321	0.719	0.855	0.956	0.368	0.713	0.172
S2	0.673	1	0.76	0.123	0.696	0.794	0.908	0.938	0.81	0.96	0.803	0.953	0.859	0.929	0.388	0.943	0.961	0.917	0.957	0.955	0.896	0.271	0.946	0.468	0.655	0.551	0.923	0.253
S3	0.614	0.76	1	0.173	0.746	0.776	0.816	0.709	0.614	0.783	0.905	0.682	0.846	0.826	0.379	0.673	0.688	0.624	0.746	0.711	0.745	0.306	0.716	0.542	0.578	0.637	0.737	0.302
S4	0.105	0.123	0.173	1	0.145	0.127	0.144	0.104	0.105	0.126	0.161	0.101	0.131	0.125	0.106	0.104	0.102	0.088	0.114	0.108	0.122	0.099	0.108	0.113	0.099	0.147	0.109	0.127
S5	0.916	0.696	0.746	0.145	1	0.574	0.615	0.618	0.794	0.669	0.926	0.636	0.753	0.696	0.484	0.616	0.64	0.667	0.631	0.666	0.672	0.409	0.697	0.902	0.924	0.444	0.713	0.248
S6	0.548	0.794	0.776	0.127	0.574	1	0.928	0.849	0.659	0.897	0.749	0.844	0.841	0.885	0.399	0.798	0.795	0.725	0.868	0.811	0.888	0.326	0.832	0.395	0.464	0.668	0.825	0.242
S7	0.6	0.908	0.816	0.144	0.615	0.928	1	0.919	0.744	0.97	0.8	0.94	0.86	0.97	0.377	0.912	0.916	0.863	0.952	0.92	0.95	0.279	0.934	0.378	0.527	0.667	0.923	0.234
S8	0.611	0.938	0.709	0.104	0.618	0.849	0.919	1	0.83	0.963	0.758	0.966	0.874	0.946	0.345	0.965	0.971	0.925	0.982	0.957	0.881	0.245	0.96	0.366	0.578	0.525	0.956	0.229
S9	0.843	0.81	0.614	0.105	0.794	0.659	0.744	0.83	1	0.808	0.815	0.858	0.791	0.839	0.497	0.874	0.853	0.907	0.828	0.881	0.779	0.443	0.885	0.644	0.813	0.471	0.894	0.222
S10	0.651	0.96	0.783	0.126	0.669	0.897	0.97	0.963	0.808	1	0.812	0.972	0.884	0.976	0.41	0.951	0.968	0.921	0.983	0.966	0.953	0.299	0.972	0.437	0.607	0.624	0.956	0.244
S11	0.837	0.803	0.905	0.161	0.926	0.749	0.8	0.758	0.815	0.812	1	0.761	0.871	0.851	0.509	0.745	0.759	0.75	0.779	0.785	0.789	0.412	0.804	0.763	0.813	0.585	0.827	0.276
S12	0.669	0.953	0.682	0.101	0.636	0.844	0.94	0.966	0.858	0.972	0.761	1	0.837	0.956	0.389	0.98	0.982	0.967	0.982	0.98	0.921	0.283	0.983	0.408	0.621	0.538	0.968	0.202
S13	0.661	0.859	0.846	0.131	0.753	0.841	0.86	0.874	0.791	0.884	0.871	0.837	1	0.917	0.426	0.819	0.832	0.779	0.865	0.838	0.83	0.349	0.856	0.575	0.676	0.528	0.887	0.287
S14	0.686	0.929	0.826	0.125	0.696	0.885	0.97	0.946	0.839	0.976	0.851	0.956	0.917	1	0.381	0.944	0.949	0.917	0.97	0.958	0.936	0.298	0.965	0.469	0.642	0.603	0.966	0.245
S15	0.359	0.388	0.379	0.106	0.484	0.399	0.377	0.345	0.497	0.41	0.509	0.389	0.426	0.381	1	0.333	0.37	0.397	0.355	0.406	0.462	0.784	0.428	0.502	0.364	0.501	0.433	0.192
S16	0.662	0.943	0.673	0.104	0.616	0.798	0.912	0.965	0.874	0.951	0.745	0.98	0.819	0.944	0.333	1	0.979	0.959	0.978	0.983	0.891	0.242	0.968	0.371	0.609	0.522	0.947	0.208
S17	0.653	0.961	0.688	0.102	0.64	0.795	0.916	0.971	0.853	0.968	0.759	0.982	0.832	0.949	0.37	0.979	1	0.972	0.981	0.98	0.903	0.258	0.981	0.402	0.625	0.524	0.965	0.213
S18	0.713	0.917	0.624	0.088	0.667	0.725	0.863	0.925	0.907	0.921	0.75	0.967	0.779	0.917	0.397	0.959	0.972	1	0.939	0.966	0.863	0.31	0.97	0.455	0.686	0.479	0.961	0.183
S19	0.635	0.957	0.746	0.114	0.631	0.868	0.952	0.982	0.828	0.983	0.779	0.982	0.865	0.97	0.355	0.978	0.981	0.939	1	0.977	0.913	0.259	0.971	0.381	0.587	0.575	0.956	0.232
S20	0.688	0.955	0.711	0.108	0.666	0.811	0.92	0.957	0.881	0.966	0.785	0.98	0.838	0.958	0.406	0.983	0.98	0.966	0.977	1	0.915	0.307	0.98	0.446	0.649	0.556	0.959	0.232
S21	0.682	0.896	0.745	0.122	0.672	0.888	0.95	0.881	0.779	0.953	0.789	0.921	0.83	0.936	0.462	0.891	0.903	0.863	0.913	0.915	1	0.339	0.943	0.493	0.613	0.649	0.906	0.235
S22	0.321	0.271	0.306	0.099	0.409	0.326	0.279	0.245	0.443	0.299	0.412	0.283	0.349	0.298	0.784	0.242	0.258	0.31	0.259	0.307	0.339	1	0.315	0.444	0.328	0.597	0.349	0.193
S23	0.719	0.946	0.716	0.108	0.697	0.832	0.934	0.96	0.885	0.972	0.804	0.983	0.856	0.965	0.428	0.968	0.981	0.97	0.971	0.98	0.943	0.315	1	0.483	0.68	0.559	0.983	0.22
S24	0.855	0.468	0.542	0.113	0.902	0.395	0.378	0.366	0.644	0.437	0.763	0.408	0.575	0.469	0.502	0.371	0.402	0.455	0.381	0.446	0.493	0.444	0.483	1	0.916	0.303	0.493	0.203
S25	0.956	0.655	0.578	0.099	0.924	0.464	0.527	0.578	0.813	0.607	0.813	0.621	0.676	0.642	0.364	0.609	0.625	0.686	0.587	0.649	0.613	0.328	0.68	0.916	1	0.278	0.688	0.182
S26	0.368	0.551	0.637	0.147	0.444	0.668	0.667	0.525	0.471	0.624	0.585	0.538	0.528	0.603	0.501	0.522	0.524	0.479	0.575	0.556	0.649	0.597	0.559	0.303	0.278	1	0.548	0.493
S27	0.713	0.923	0.737	0.109	0.713	0.825	0.923	0.956	0.894	0.956	0.827	0.968	0.887	0.966	0.433	0.947	0.965	0.961	0.956	0.959	0.906	0.349	0.983	0.493	0.688	0.548	1	0.213
S28	0.172	0.253	0.302	0.127	0.248	0.242	0.234	0.229	0.222	0.244	0.276	0.202	0.287	0.245	0.192	0.208	0.213	0.183	0.232	0.232	0.235	0.193	0.22	0.203	0.182	0.493	0.213	1
RSI	0.769	0.913	0.81	0.297	0.803	0.84	0.904	0.895	0.885	0.933	0.894	0.912	0.888	0.935	0.545	0.895	0.906	0.892	0.915	0.924	0.909	0.478	0.935	0.622	0.737	0.695	0.932	0.398

*Rheum officinale Bail.*: S1, S5, S8, S11, S13, S14, S16, S17, S21, S24, S25, S26, S27, S28; *Rheum palmatum* L.: S2, S6, S7, S10, S18, S19, S23; *Rheum tanguticum* Maxim. ex Balf.: S3, S4, S9, S12, S15, S20, S22; All the rhubarb samples were examined to create a mean global chromatogram as the standard fingerprint, and the reference similarity index (RSI) of each chromatogram of rhubarb samples against the mean global chromatogram was calculated using the Similarity Evaluation System for Chromatographic Fingerprint of TCM (version 2004). Group A includes sample S2, S7–S14, S16–S21, S23 and S27 (RSI ≥ 0.885; The RSI values of these samples are approximate or higher than 0.9); Group B includes sample S1, S3–S6, S15, S22, S24–S26 and S28 (RSI ≤ 0.84; The RSI values of most of these samples are far less than 0.9)

### DL measurements of the 28 commercial rhubarb samples

**Fig. 4:**
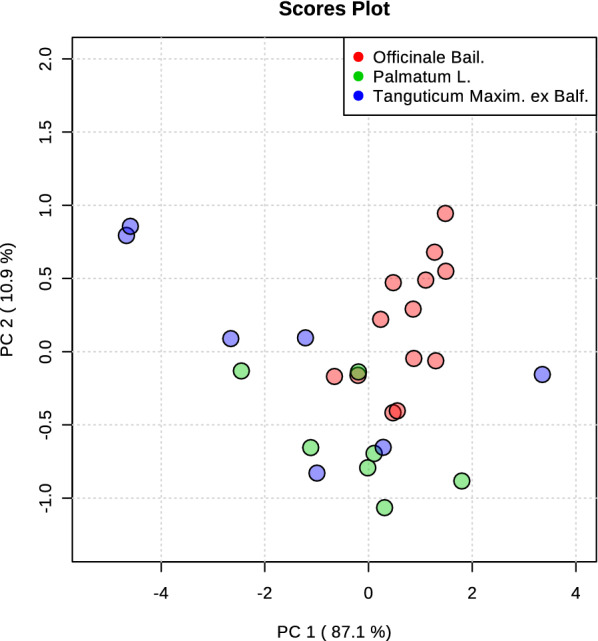
PCA scores obtained from the DL data. PCA score plots of the DL properties obtained from all batches of commercial rhubarb samples

**Fig. 5:**
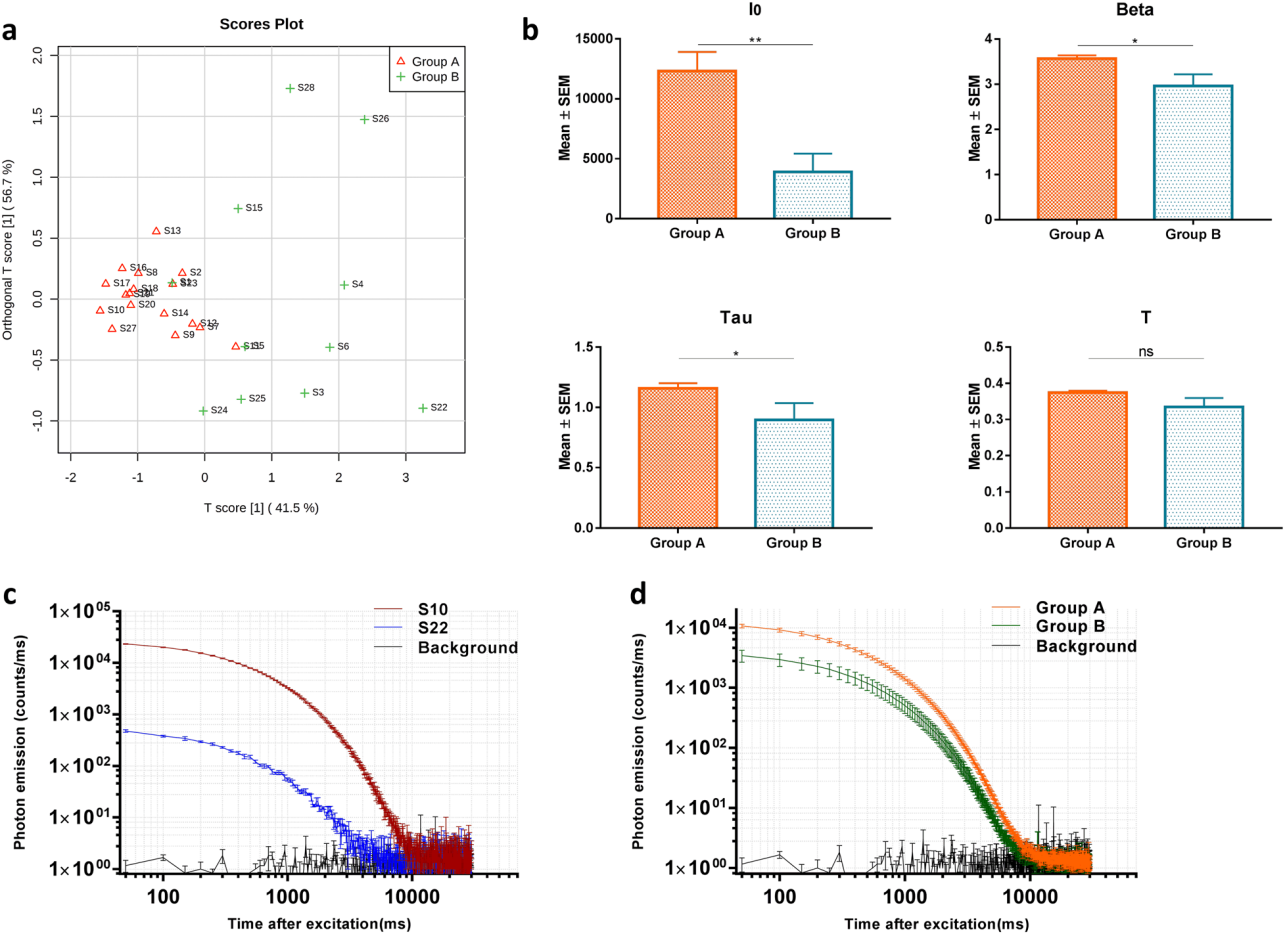
DL analysis of the commercial rhubarb samples. **a** OPLS-DA score plots of the DL properties obtained from all batches of commercial rhubarb samples with cross-validation revealed predictive accuracy of 0.527 (Q^2^) and goodness-of-fit of 0.608 (R^2^), respectively; **b** Histograms comparing the DL properties between two groups. *p < 0.05; **p < 0.01; ns, no significant difference (two-tailed, unpaired Student’s t-test). Beta is an index factor associated with the rate of DL decay, I0 is the initial intensity of the DL curve, and T and Tau represents the decay time and DL characteristics, respectively. **c** DL decay curves of commercial rhubarb sample S10 and S22; **d** DL decay curves for pooled rhubarb samples from group **a** and group **b**. Data are plotted as the mean ± SEM. Note that the data are plotted on a log–log scale

DL measurements were applied to the 28 commercial rhubarb samples. To calculate the four properties of the DL curves, a hyperbolic function was used to fit the observed decay curves (Additional file 1: Table S4). Next, PCA was used to obtain a focused view of the variance in the four DL properties in order to differentiate rhubarb sub-species. However, there was no clear separation (Fig. 4). To evaluate whether DL properties can be used to create a similar quality standard as compared to HPLC fingerprint analysis. OPLS-DA was carried out for the DL data using the supervised classes (Group A and Group B) obtained from fingerprint analysis (Table 2). The result illustrated that DL properties can also reasonably stratify commercial rhubarb samples between the Group A and the Group B (Fig. 5a). To analyze further the difference between DL properties, a two-tailed, unpaired Student’s t-test was used to compare the four DL properties between the Group A and the Group B. The results revealed that I0, Beta and Tau differed significantly between the two sub-groups, and the values of these DL properties in the group A are higher than that in the group B (Fig. 5b). Figure 5c, d illustrate the different DL decay curves of rhubarb samples representing the two different sub-groups.

### Correlation between identified chemical compounds and DL properties

**Fig. 6:**
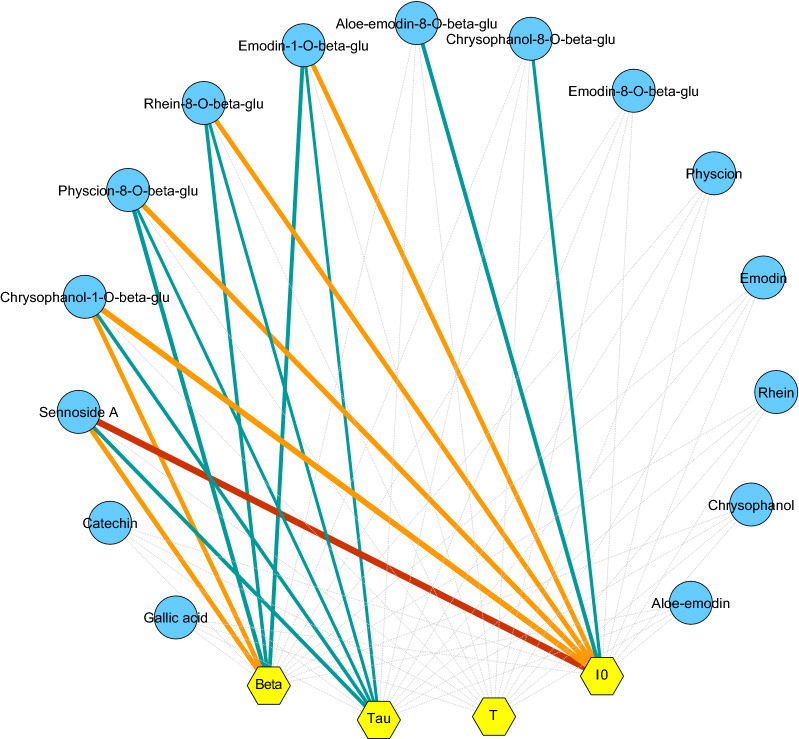
Correlation network between the chemical compounds and DL properties measured in the commercial rhubarb samples. The negative and weak correlations are indicated with blue lines (0.30 < | *ρ* | < 0.50), the negative and moderate correlations are indicated with orange lines (0.50 < | *ρ* | < 0.70) and the negative and strong correlations are indicated with red line ((|*ρ*| > 0.70). Thicker lines indicate a stronger correlation. (https://www.dummies.com/education/math/statistics/how-to-interpret-a-correlation-coefficient-r/). Non-liner correlations (| *ρ* | < 0.30) are indicated with grey lines. The length of each line has no meaning

The DL measurements and the HPLC fingerprint analysis displayed the similar identified effects in the 28 commercial rhubarb samples. Next, we determined the correlations between the DL properties and chemical compounds for all commercial rhubarb samples using the Spearman’s correlation. We found negative correlations (| *ρ* | > 0.30) between DL properties and chemical compounds (Additional file 1: Table S5). These correlations are depicted visually in Fig. 6.

### Validation tests using wild rhubarb materials

**Fig. 7:**
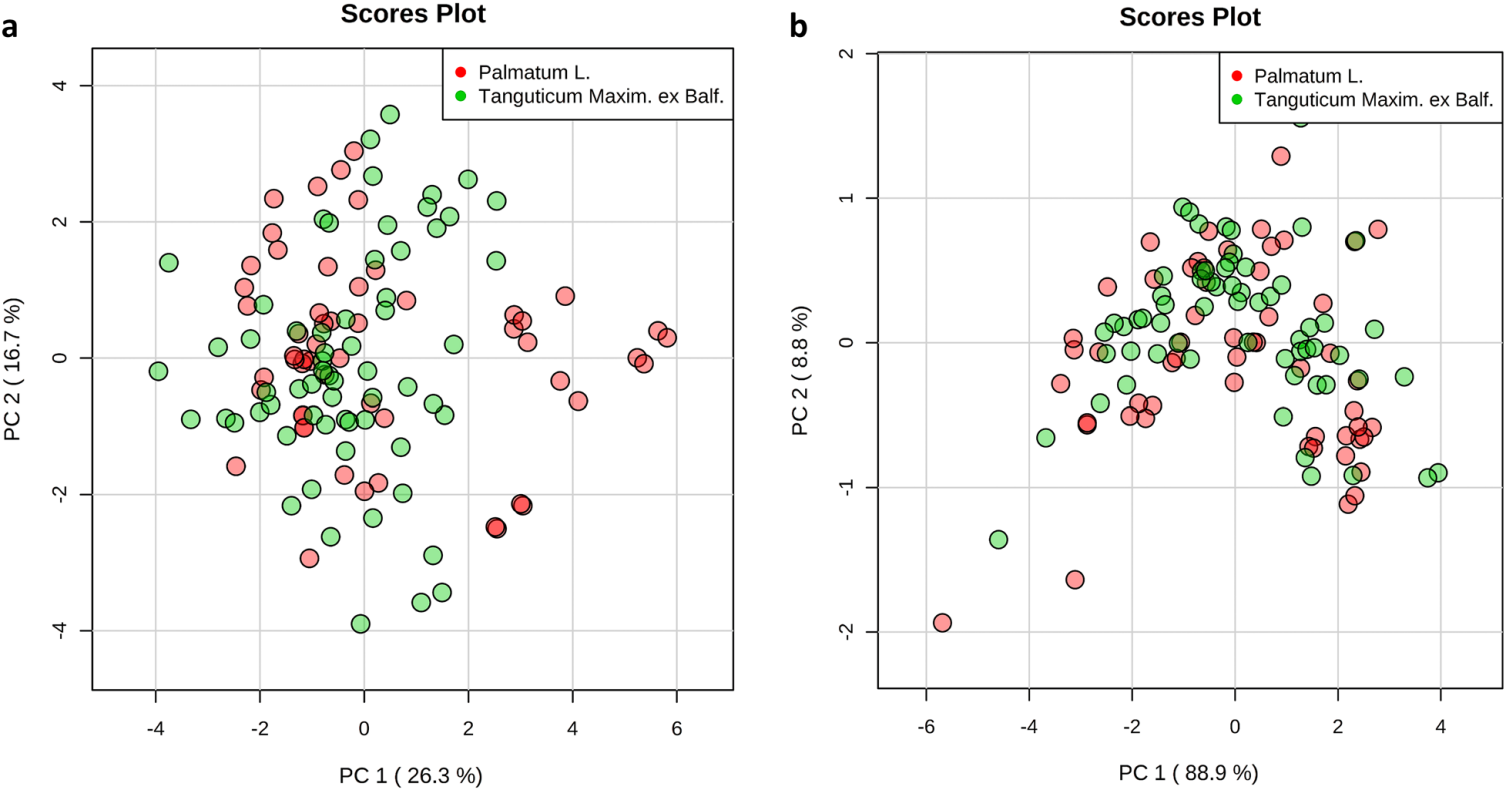
PCA scores of wild rhubarb samples. **a** PCA score plots of the contents of the identified chemical compounds obtained from all batches of wild rhubarb samples. The identified compounds were almost same with the compounds tested in the HPLC analysis of commercial rhubarb samples [14]; **b** PCA score plots of the DL properties obtained from all batches of wild rhubarb samples

**Fig. 8:**
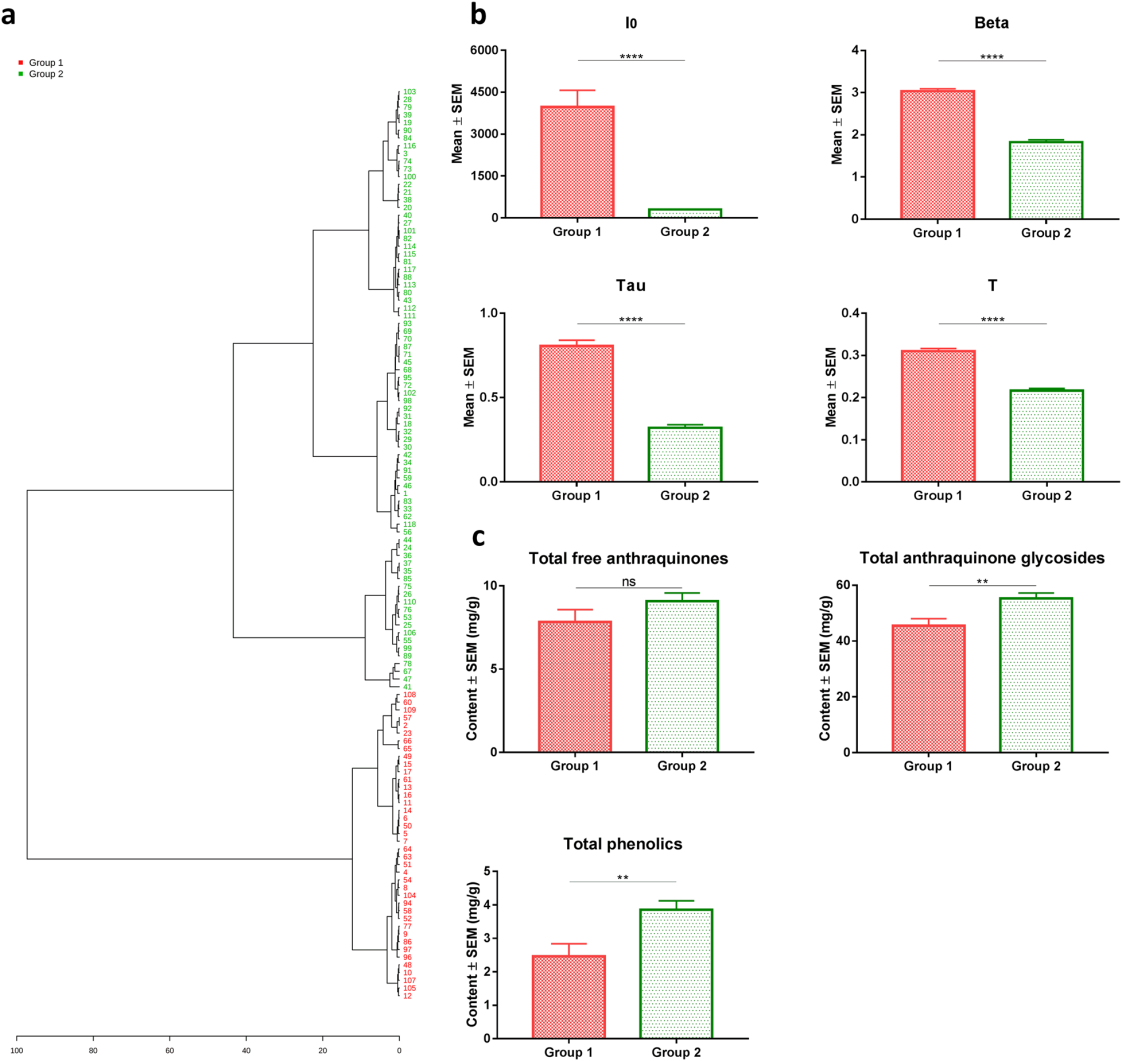
DL analysis and chemical analysis of the wild rhubarb samples. **a** Dendrogram of the hierarchical cluster analysis of DL properties in the wild rhubarb samples analysis (Distance Measure: Euclidean; Clustering Algorithm: Ward). **b** Histograms comparing the DL properties between two groups. ****p < 0.0001 (two-tailed, unpaired Student’s t-test). **c** Histograms comparing the contents of chemical components between two groups. **p < 0.01; ns, no significant difference (two-tailed, unpaired Student’s t-test)

In the tests of the commercial rhubarb samples, we found that both HPLC fingerprint analysis and DL measurements were not able to distinguish sub-species of rhubarb materials. In addition, the commercial rhubarb samples with low level of glycoside-containing component usually possessed high value of DL properties (e.g., Group A in Figs. 3, 5). To validate those results, both chemical analysis and DL measurements were used to study 118 batches of wild rhubarb samples (55 batches of *Rheum palmatum* L. and 63 batches of *Rheum tanguticum* Maxim. ex Balf.). PCA analysis was performed to distinguish rhubarb sub-species using the data of identified individual chemical compounds and DL properties, respectively. The results showed that both chemical data and DL data cannot stratify that two sub-species successfully (Fig. 7). Next, the unsupervised hierarchical cluster analysis was used to classify wild rhubarb samples into two groups (Fig. 8a). A two-tailed, unpaired Student’s t-test was used to compare the four DL properties between these two groups. The results revealed that all the DL properties differed significantly between the two groups. Then, we named the group with high value of DL properties as group 1, and the other group as group 2, in order to distinguish from group A and group B in the tests of the commercial rhubarb samples. Figure 8b illustrates that the values of DL properties in group 1 were higher than that in group 2. But the total amounts of glycoside-containing compounds and polyphenol compounds in group 1 were significantly lower than that in group 2 (Fig. 8c). This results validated basically the outcomes in the tests of the commercial rhubarb samples.

### The results of cathartic activity tests

According to the fingerprint analysis of the commercial rhubarb samples, we found that the significant differences in chemical composition such as glycoside-containing compounds between group A and B (Fig. 3c, d). Based on the concept of Chinese herbal medicine (Fig. 1), rhubarb has cathartic activity. It has been reported that the cathartic activity depends mainly on the presence of glycoside-containing compounds such as anthraquinone glycosides and sennosides [24, 25]. Therefore, we questioned whether group A and group B would have different cathartic activity. To evaluate the cathartic activity of rhubarb samples, mice model was used. In this study, commercial rhubarb sample S10 and S22 which represented group A and B, respectively, were selected. As the contents of glycoside-containing compounds differed significantly between these two samples (*p *< 0.05), but there was no significant difference in free anthraquinones and polyphenol compounds (*p *> 0.05).

Mice were treated using rhubarb extract solution, then the excretion status of mice was recorded. Almost all the using dosages of S10 and S22 could significantly decrease the incubation period of defecation compared to the control group (Table 3). S10 in low dosage group did not show the significant difference in incubation period compared to the control group. However, the purgative effect of S22 in low dosage group was significantly better than that of S10 (Table 3). All three dosage groups of S22 showed a significant increase in terms of quantity and weight of feces compared to the control group. However, S10 showed similar activities only at the high concentrations. In addition, in the low concentration group, there was a significant difference between S22 and S10 in terms of feces weight (Table 3). Moreover, both S10 and S22 did not lead to diarrhea in mice at the low concentration. Both the medium and high concentration groups of S10 and S22 can result in diarrhea, and S22 caused significantly increase in diarrhea rate compared to the control group (Table 4). In the test of small intestine propelling, almost all the using dosages of S10 and S22 could significantly increase the intestine propelling rate compared to the control group, except S10 at the low concentration (Table 5). As the most activities of S22 at low dosage were better than that of S10 at the same concentration, as well as the higher diarrhea rate and more feces in both medium and high concentration group of S22. We considered S22 has better purgative effect compared to S10.

**Table 3:** Excretion status of mice after taking rhubarb decoction ( ± *sd*)

Group	Dosage (g crude rhubarb/kg)	*n*	Charcoal powder-containing feces		
			Incubation period (min)	Number	Weight (mg)
Control	–	8	280.5 ± 36.1	0.4 ± 0.7	2.0 ± 3.7
S10-Low	1.0	8	255.4 ± 62.4	1.1 ± 1.6	5.8 ± 8.9
S10-Medium	2.5	8	148.4 ± 65.5***	5.3 ± 4.1	25.5 ± 16.3
S10-High	5.0	9	105.5 ± 44.3***	8.0 ± 2.9**	65.7 ± 25.0**
S22-Low	1.0	8	133.7 ± 60.7***^###^	5.1 ± 2.5*	59.0 ± 30.9*^#^
S22- Medium	2.5	8	129.8 ± 40.3***	6.9 ± 2.7**	86.6 ± 50.6^*^
S22-High	5.0	9	88.9 ± 21.0***	6.9 ± 1.8***	62.3 ± 22.3**

Comparison with control group: *p < *0.05*; **p < *0.01*; ***p < *0.001*; Comparisons with the same dose of S10: ^*#*^*p *< *0.05*; ^*###*^*p *< *0.001*

**Table 4:** Diarrhea rate of mice after taking rhubarb decoction

Group	Dosage (g crude rhubarb/kg)	*n*	The mice of loose stools (n)	Diarrhea rate (%)
Control	–	8	0	0
S10-Low	1.0	8	0	0
S10-Medium	2.5	8	1	12.5
S10-High	5.0	9	3	33.3
S22-Low	1.0	8	0	0
S22- Medium	2.5	8	5	62.5*
S22-High	5.0	9	8	88.9***^#^

Comparison with control group: *p < 0.05; ***p < 0.001. Comparisons with the same dose of S10: ^#^*p *< 0.05

**Table 5:** Small intestine propelling rate of mice after taking rhubarb decoction

Group	Dosage (g crude rhubarb/kg)	*n*	Length 1 (cm)	Length 2 (cm)	Charcoal powder propelling( %)
Control	–	8	26.7 ± 5.1	43.9 ± 3.3	60.6 ± 9.9
S10-Low	1.0	8	35.3 ± 4.0	46.6 ± 1.8	75.6 ± 8.1
S10-Medium	2.5	9	35.7 ± 2.6	43.9 ± 2.0	81.3 ± 5.8**
S10-High	5.0	9	35.6 ± 2.5	43.8 ± 2.8	81.1 ± 1.0*
S22-Low	1.0	8	34.4 ± 2.4	43.5 ± 2.6	79.2 ± 6.3*
S22-Medium	2.5	9	35.0 ± 2.2	42.6 ± 2.9	82.4 ± 6.1**
S22-High	5.0	9	35.3 ± 2.1	44.1 ± 2.3	80.1 ± 1.6*

Comparison with control group: *p < 0.05; **p < 0.01. Length 1 indicates the distance from pylorus to the front end of charcoal powder; Length 2 indicates the total length of small intestine

## Discussion

Our preliminary results demonstrated that chemical analysis and DL measurements were not able to segregate of rhubarb samples (both commercial and wild samples) according to difference in sub-species. Ge et al. reported the same results using PCA to analyze the ^1^H NMR data of different rhubarb sub-species samples [33]. This data may support the idea why in Chinese pharmacopeia, different sub-species were collected because of their similarity in chemical composition thought with difference sub-species. Our results suggested that DL properties of rhubarb samples may be more relevant to herb’s chemical profiles. Many studies have proved that the variability of DL is closely related to the chemical structure of samples [14–16, 34, 35]. This is because of the changes of the conformation and structure of sample’s molecule can affect the behavior of luminescence [36]. In this research, DL measurements show the similar results in terms of distinguishing the commercial rhubarb samples compared with HPLC fingerprint analysis. It indicates that DL may be a complementary tool to build up a rapid and cheap measurement in assessment the quality of herbal medicine. In addition, we found that the values of DL properties were lower when the content of glycoside-containing compounds was higher in the commercial rhubarb samples, and the DL properties have the negative and liner correlations with glycoside-containing compounds (Fig. 6; Additional file 1: Table S5). This result is in principle consistent with our previous results of ginsenoside extracts [37]. It indicates that DL may be closely related to specific class of chemical constituents in herbs. Therefore, a validation test was designed using wild rhubarb materials. Although different HPLC methods were used for the test of the wild rhubarb samples, the identified compounds [14] were almost same with the compounds tested in the HPLC analysis of commercial rhubarb samples. And the negative relations between DL properties and glycoside-containing compounds were presence again in the wild rhubarb samples (Fig. 8). Our results demonstrated that the validation test supports the identified correlations. Moreover, the negative relation between DL properties and glycoside-containing compounds were observed. Investigating the chemical component which closely related to glycoside-containing compounds may further help to find out the direct chemical targets of DL in herbal quality control. Next, in the test of wild rhubarb samples, we found that the content of polyphenol compounds (catechin and gallic acid) also has the response to the changes of DL properties. Wild rhubarb samples grow usually at very different altitude localities [14], and the altitude is related to intensity of solar radiation and fluctuations in ambient temperature [38, 39]. These environmental factors can lead to the accumulation of polyphenol compounds in plants [40, 41]. Therefore, the polyphenol compounds in wild rhubarb samples may demonstrate more variations which interacts to the changes of DL properties. It indicates that DL, may correspond to multi-chemical components in herbs. Therefore, to build up DL based herbal quality assessment tool, the comprehensive metabolic profile of herbal materials should be involved in the future research, and the influence of environmental factors should be taken into consideration.

HPLC fingerprint has been accepted as one of the most important approaches for quality control of herbs [42]. In this research, the standard HPLC chromatographic fingerprint was developed with the median of chromatograms of all commercial rhubarb samples, then the reference similarity index was calculated between individual fingerprint and standard fingerprint. Some commercial rhubarb samples were found very low similarity index (e.g., S22) compared to the standard chromatographic fingerprint, and the reason can attribute to the higher contents of glycoside-containing compounds. Therefore, representative of two sub-groups of rhubarb sample S10 (Group A) and S22 (Group B) have been used to study cathartic activity in mice model. The results showed clearly the cathartic activity of S22 was better than that of S10. According to chemical and biological studies, the presence of glycoside-containing component contribute to the cathartic activity of rhubarb materials [24, 25]. Our results are consistent with the previous studies. Therefore, the relative low similarity in fingerprint may not indicate the poor bio-activities of herbs. At present, the quality control of herbs is based on the determination of index components and fingerprint patterns. For instance, according to the 2020 Chinese Pharmacopoeia, the total amount of free anthraquinone derivatives (e.g. aloe-emodin, rhein and emodin etc.) defines the quality of rhubarb materials [22]. However, S10 and S22 have the similar level of free anthraquinone, but show different cathartic activity, it demonstrated again that quality control based on individual chemical components may not represent the total quality. Biological activities are the most relevant quality control indicators, and many herbs possess multi-bioactivities. Therefore, although bioactivity based herbal quality assessment should be taken into consideration, the various biological activities is the challenges to assessment which should be addressed in the future work. For example, rhubarb materials can be classified based on the different bio-activities according to the amounts of specific active chemical components. And the HPLC fingerprint and DL measurements could be used to identify characteristic properties for rhubarb materials based on the specific bio-activity in order to guide the evaluation of quality. It will be benefit for the therapeutic application of herbs, as well as the quality control on the market. This integrated assessment strategy may be a new direction, and promote development of medicinal herbs.

## Conclusion

In this research, we found that there was no significant difference of chemical fingerprints and DL signals among the different species of medicinal rhubarb. In addition, DL measurements show the similar results in terms of distinguishing the rhubarb samples compared with fingerprint analysis, and DL properties can be linked to the specific chemical component which reflects rhubarb’s bioactivity. It indicates that DL is a promising method to evaluate herbal quality. Moreover, biological activities are a key representing quality. Similarity of evaluation of HPLC profiling is current used method, however linking the biological activities with chemical profiling is the solution for quality control for herbal medicine. This proof-of-concept study may provide a suitable foundation for follow-up studies. Both HPLC analysis and DL measurement have their own merit (Table 6) in assessment of herb quality. As a novel method, DL measurement may be not suitable to show clear fingerprint characteristics. In addition, the understanding of the chemical components of herbs, that are sensitive to DL measurement, is still limited. Therefore, the further research should concentrate the response between DL spectral features and specific chemical components of herbs, in particular polysaccharides and glycosides. Moreover, additional research should emphasize bioactivity based quality assessments for herbs in order to promote the shift from evaluating chemical pattern to evaluating activities corresponding to the chemical constituents. In conclusion, DL provides a technique for studying the overall property of herbal materials [43], and the integrated assessment by measuring chromatographic fingerprint and DL based on bioactivity may provide a novel means to measure herbal quality control.

**Table 6:** The advantages of HPLC fingerprint and DL measurement

Analytical method	Advantages
HPLC fingerprint	High accuracy and sensitivity, component separation, automation, wide application
DL measurement	Fast, cheap, holistic measurement, non-extraction required, pollution-free

## Supplementary information

**Additional file 1: Fig. S1.** Chromatographic fingerprinting of commercial rhubarb samples (Black line: Standard solution; Purple line: Sample solution), **Fig. S2.** HPLC chromatography of 28 batches of commercial rhubarb. “R” indicates the standard chromatography creating by Similarity Evaluation System for Chromatographic Fingerprint of TCM (version 2004)., **Table S1.** The content of identified compounds of commercial rhubarb samples, **Table S2.** The retention time of common peaks of commercial rhubarb samples, **Table S3.** The peak area of common peaks of commercial rhubarb samples, **Table S4.** The value of four DL properties of the 28 commercial rhubarb samples, **Table S5.** The correlation coefficient between DL properties and chemical compounds, **Table S6.** The value of DL properties and chemical components of the 118 wild rhubarb samples.

## Data Availability

The datasets used in this study are available from the corresponding author upon reasonable request.

## Abbreviations

DL: Delayed luminescence
HPLC: High performance liquid chromatography
PCA: Principal component analysis
OPLS-DA: Orthogonal projections to latent structures discriminant analysis
ANOVA: One-way analysis of variance
LSD: Least significant difference
NMR: Nuclear magnetic resonance
RSI: Reference similarity index
