# Supplementary material for: Integrated assessment of medicinal rhubarb by combination of delayed luminescence and HPLC fingerprint with emphasized on bioactivities based quality control
Source: Chin Med. 2020 Jul 14;15:72. doi: 10.1186/s13020-020-00352-8 (PMC7362467; doi:10.1186/s13020-020-00352-8)
Supplement: Supplementary file 1 — Additional file 1: Fig. S1. Chromatographic fingerprinting of commercial rhubarb samples (Black line: Standard solution; Purple line: Sample solution), Fig. S2. HPLC chromatography of 28 batches of commercial rhubarb. “R” indicates the standard chromatography creating by Similarity Evaluation System for Chromatographic Fingerprint of TCM (version 2004)., Table S1. The content of identified compounds of commercial rhubarb samples, Table S2. The retention time of common peaks of commercial rhubarb samples, Table S3. The peak area of common peaks of commercial rhubarb samples, Table S4. The value of four DL properties of the 28 commercial rhubarb samples, Table S5. The correlation coefficient between DL properties and chemical compounds, Table S6. The value of DL properties and chemical components of the 118 wild rhubarb samples. [file 13020_2020_352_MOESM1_ESM.docx]

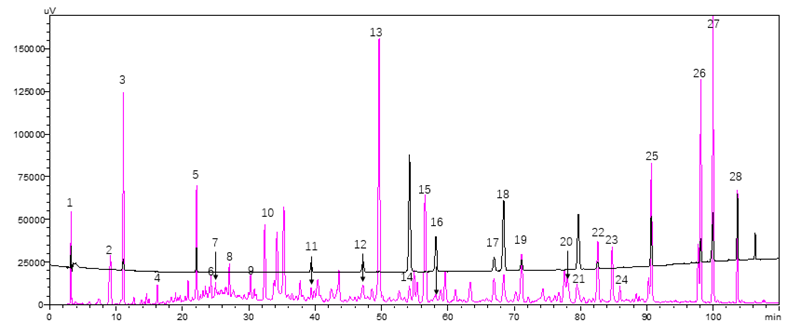


**Fig. S1. Chromatographic fingerprinting of commercial rhubarb samples (Black line: Standard solution; Purple line: Sample solution).** The peaks named No.1 to No.28 indicate common peaks of commercial rhubarb samples. No 3., Gallic acid; No 5., Catechin; No 11., Aloe-emodin-8-O-beta-D-glucoside; No 12., Rhein-8-O-beta-D-glucoside; No 14., Sennoside A; No 16., Emodin-1-O-beta-D-glucoside; No 17., Chrysophanol-1-O-beta-D-glucoside; No 18., Chrysophanol-8-O-beta-D-glucoside; No 19., Emodin-8-O-beta-D-glucoside; No 21., Physcion-8-O-beta-D-glucoside; NO 22., Aloe emodin; No 25., Rhein; No 26., Emodin; No 27., Chrysophanol; No 28., Physcion.


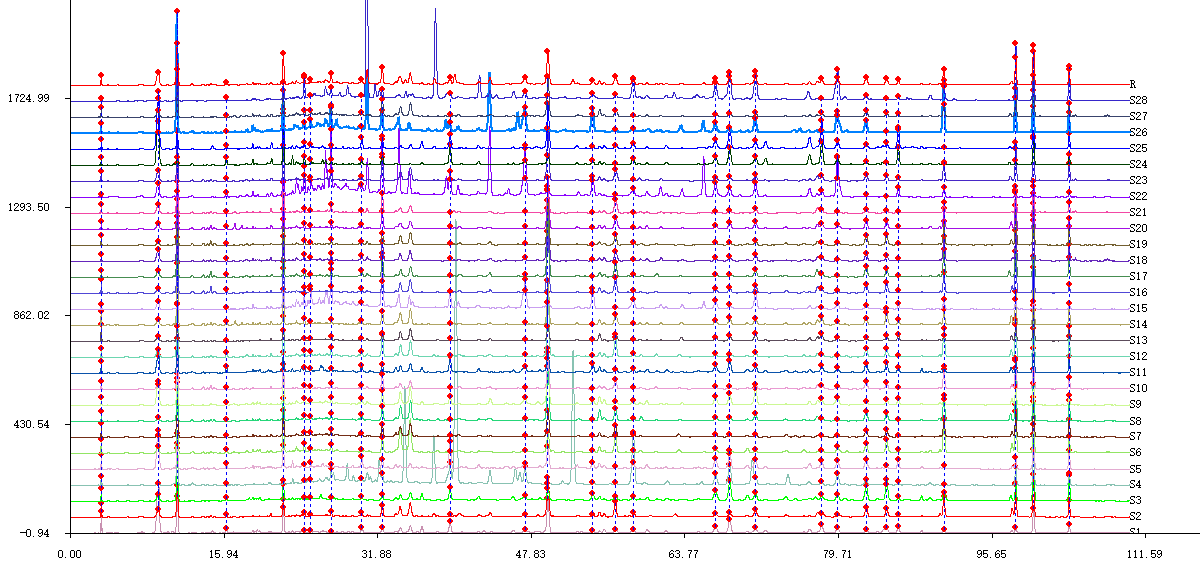


**Fig. S2. HPLC chromatography of 28 batches of commercial rhubarb.** “R” indicates the standard chromatography creating by Similarity Evaluation System for Chromatographic Fingerprint of TCM (version 2004).

Table S1 The content of identified compounds of commercial rhubarb samples

| Content（mg/g） | | | | | | | | | | | | | | | | | | | | | | | | | | | | |
| --- | --- | --- | --- | --- | --- | --- | --- | --- | --- | --- | --- | --- | --- | --- | --- | --- | --- | --- | --- | --- | --- | --- | --- | --- | --- | --- | --- | --- |
| No | S1 | S2 | S3 | S4 | S5 | S6 | S7 | S8 | S9 | S10 | S11 | S12 | S13 | S14 | S15 | S16 | S17 | S18 | S19 | S20 | S21 | S22 | S23 | S24 | S25 | S26 | S27 | S28 |
| Gallic acid | 5.22 | 4.39 | 7.57 | 7.27 | 3.62 | 9.05 | 7.66 | 5.94 | 4.52 | 5.72 | 4.89 | 7.51 | 2.22 | 8.61 | 0.65 | 7.82 | 7.6 | 6.39 | 6.36 | 6.61 | 6.96 | 0.74 | 7.19 | 0.96 | 2.52 | 17.17 | 4.81 | 4.29 |
| Catechin | 7.11 | 4.91 | 4.45 | 0.94 | 6.68 | 11.99 | 7.45 | 6.53 | 9.93 | 6.69 | 5.73 | 11.38 | 2.93 | 10.08 | 13.83 | 7.11 | 11.58 | 14.86 | 7.26 | 9.42 | 8.84 | 24.59 | 10.19 | 11.26 | 9.93 | 11.48 | 7.88 | 4.8 |
| Aloe-emodin-8-O-beta-D-glucoside | 0.91 | 0.12 | 1 | 1.65 | 2.22 | 0.06 | 0.03 | 0.07 | 0.59 | 0.07 | 1.22 | 0.06 | 0.17 | 0.1 | 1.27 | 0.05 | 0.07 | 0.13 | 0.05 | 0.1 | 0.03 | 1.24 | 0.09 | 1.59 | 1.18 | 0.46 | 0.14 | 0.07 |
| Rhein-8-O-beta-D-glucoside | 0.59 | 0.57 | 1.51 | 3.21 | 1.48 | 0.44 | 0.29 | 0.37 | 1.66 | 0.4 | 2.02 | 0.45 | 0.35 | 0.34 | 8.68 | 0.51 | 0.51 | 0.65 | 0.28 | 0.42 | 0.16 | 12.92 | 0.31 | 1.68 | 0.4 | 6.39 | 0.47 | 1.5 |
| Sennoside A | 0.65 | 0.55 | 1.66 | 0.37 | 1.42 | 0.56 | 0.38 | 0.32 | 1.29 | 0.12 | 2 | 0.25 | 0.22 | 0.15 | 4.96 | 0.09 | 0.15 | 0.28 | 0.1 | 0.14 | 0.05 | 4.75 | 0.17 | 1.06 | 0.51 | 5.89 | 0.5 | 1.38 |
| Emodin-1-O-beta-D-glucoside | 0.39 | 0.49 | 0.38 | 5.94 | 0.67 | 0.3 | 0.24 | 0.27 | 0.47 | 0.15 | 0.39 | 0.32 | 0.19 | 0.1 | 1.22 | 0.29 | 0.3 | 0.14 | 0.33 | 0.09 | 0.06 | 0.96 | 0.14 | 0.75 | 0.63 | 0.4 | 0.1 | 2.29 |
| Chrysophanol-1-O-beta-D-glucoside | 2.26 | 1.15 | 3.57 | 3.66 | 3.51 | 1.29 | 0.88 | 1.11 | 3.11 | 0.74 | 2.73 | 0.85 | 1.27 | 1.29 | 2.69 | 1.38 | 0.76 | 0.87 | 0.82 | 1.16 | 0.5 | 5.22 | 1.04 | 2.89 | 2.98 | 3.13 | 0.84 | 4.68 |
| Chrysophanol-8-O-beta-D-glucoside | 28.37 | 10.25 | 42.42 | 45.42 | 33.45 | 7.23 | 5.65 | 7.97 | 24.62 | 4.25 | 27.17 | 6.07 | 11.79 | 15.76 | 9.59 | 11.74 | 8.58 | 11.22 | 6.11 | 15.62 | 5.35 | 22.29 | 10.17 | 36.63 | 44.53 | 10.71 | 8.01 | 62.52 |
| Emodin-8-O-beta-D-glucoside | 0.75 | 0.99 | 1.01 | 0.12 | 1.27 | 0.38 | 0.28 | 0.95 | 2.32 | 0.63 | 0.92 | 0.6 | 0.97 | 0.9 | 2.34 | 1.08 | 1.04 | 0.88 | 0.58 | 0.99 | 0.62 | 2.13 | 0.83 | 1.21 | 1.16 | 1.89 | 0.53 | 3.97 |
| Physcion-8-O-beta-D-glucoside | 0.7 | 0.44 | 0.81 | 1.46 | 1.11 | 0.87 | 0.22 | 0.23 | 1.12 | 0.35 | 0.62 | 0.28 | 0.5 | 0.43 | 0.82 | 0.4 | 0.14 | 0.39 | 0.4 | 0.67 | 0.22 | 5.08 | 0.32 | 0.89 | 1 | 3.12 | 0.23 | 4.5 |
| Aloe emodin | 0.62 | 1.71 | 2.76 | 1.07 | 1.82 | 1.74 | 1.22 | 1.83 | 0.72 | 1.43 | 1.84 | 1.6 | 0.75 | 1.34 | 0.7 | 1.63 | 1.93 | 1.44 | 1.85 | 1.61 | 0.68 | 0.36 | 1.18 | 0.72 | 0.8 | 2.32 | 1.16 | 0.71 |
| Rhein | 3.81 | 24.02 | 14.29 | 1.36 | 3.73 | 41.79 | 20.86 | 31.85 | 6.44 | 24.11 | 7.48 | 35.5 | 6.46 | 17.94 | 12.11 | 24.4 | 34.04 | 28.18 | 28.54 | 25.08 | 13.17 | 11.56 | 22.28 | 0.99 | 1.01 | 66.13 | 15.12 | 17.17 |
| Emodin | 0.23 | 1.41 | 0.82 | 0.87 | 0.5 | 2.13 | 1.11 | 2.49 | 0.82 | 1.41 | 0.55 | 1.74 | 0.75 | 1.53 | 0.45 | 1.83 | 2.26 | 1.23 | 1.87 | 1.78 | 1.23 | 0.2 | 1.52 | 0.2 | 0.31 | 2.15 | 0.92 | 2.72 |
| Chrysophanol | 1.46 | 2.48 | 3.88 | 2.07 | 2.6 | 2.98 | 2.32 | 3.06 | 1.29 | 2.43 | 2.61 | 2.75 | 2.08 | 3.39 | 0.49 | 2.11 | 3.39 | 2.63 | 2.42 | 2.1 | 1.85 | 0.66 | 2.59 | 1.58 | 2.51 | 2.5 | 2.47 | 2.89 |
| Physcion | 7.18 | 13.77 | 20.99 | 9.64 | 14.98 | 13.53 | 14.3 | 16.47 | 7.48 | 9.24 | 13.32 | 16.65 | 12.13 | 17.81 | 4.23 | 7.11 | 15.89 | 13.07 | 14.67 | 8.32 | 8.3 | 3.63 | 14.13 | 8.17 | 12.18 | 16.96 | 10.64 | 27.09 |

Table S2 The retention time of common peaks of commercial rhubarb samples

| The ID of commercial rhubarb samples | | | | | | | | | | | | | | | | | | | | | | | | | | | | |
| --- | --- | --- | --- | --- | --- | --- | --- | --- | --- | --- | --- | --- | --- | --- | --- | --- | --- | --- | --- | --- | --- | --- | --- | --- | --- | --- | --- | --- |
| Peak No. | S1 | S2 | S3 | S4 | S5 | S6 | S7 | S8 | S9 | S10 | S11 | S12 | S13 | S14 | S15 | S16 | S17 | S18 | S19 | S20 | S21 | S22 | S23 | S24 | S25 | S26 | S27 | S28 |
| 1 | 0.064 | 0.064 | 0.064 | 0.065 | 0.064 | 0.064 | 0.064 | 0.064 | 0.064 | 0.064 | 0.064 | 0.064 | 0.064 | 0.064 | 0.065 | 0.064 | 0.064 | 0.064 | 0.064 | 0.064 | 0.064 | 0.065 | 0.064 | 0.064 | 0.064 | 0.065 | 0.064 | 0.065 |
| 2 | 0.184 | 0.185 | 0.184 | 0.186 | 0.185 | 0.185 | 0.185 | 0.184 | 0.185 | 0.185 | 0.184 | 0.185 | 0.184 | 0.184 | 0.186 | 0.185 | 0.184 | 0.185 | 0.184 | 0.184 | 0.184 | 0.186 | 0.184 | 0.184 | 0.184 | 0.186 | 0.184 | 0.188 |
| 3 | 0.223 | 0.224 | 0.223 | 0.225 | 0.224 | 0.223 | 0.223 | 0.223 | 0.223 | 0.223 | 0.223 | 0.223 | 0.223 | 0.223 | 0.226 | 0.223 | 0.223 | 0.224 | 0.223 | 0.223 | 0.223 | 0.225 | 0.223 | 0.223 | 0.223 | 0.225 | 0.223 | 0.226 |
| 4 | 0.326 | 0.327 | 0.326 | 0.328 | 0.327 | 0.326 | 0.326 | 0.326 | 0.327 | 0.326 | 0.326 | 0.326 | 0.326 | 0.326 | 0.329 | 0.326 | 0.326 | 0.327 | 0.326 | 0.326 | 0.326 | 0.329 | 0.326 | 0.326 | 0.326 | 0.328 | 0.326 | 0.33 |
| 5 | 0.445 | 0.446 | 0.445 | 0.449 | 0.446 | 0.445 | 0.445 | 0.445 | 0.445 | 0.445 | 0.445 | 0.445 | 0.445 | 0.445 | 0.449 | 0.446 | 0.445 | 0.445 | 0.445 | 0.445 | 0.445 | 0.449 | 0.445 | 0.445 | 0.445 | 0.449 | 0.445 | 0.449 |
| 6 | 0.489 | 0.489 | 0.489 | 0.492 | 0.49 | 0.489 | 0.489 | 0.489 | 0.489 | 0.489 | 0.489 | 0.489 | 0.489 | 0.489 | 0.493 | 0.489 | 0.489 | 0.49 | 0.489 | 0.489 | 0.489 | 0.493 | 0.489 | 0.489 | 0.489 | 0.493 | 0.489 | 0.491 |
| 7 | 0.503 | 0.503 | 0.502 | 0.507 | 0.503 | 0.503 | 0.503 | 0.503 | 0.503 | 0.503 | 0.503 | 0.503 | 0.503 | 0.503 | 0.507 | 0.503 | 0.503 | 0.503 | 0.503 | 0.503 | 0.503 | 0.507 | 0.503 | 0.502 | 0.503 | 0.507 | 0.503 | 0.507 |
| 8 | 0.545 | 0.546 | 0.545 | 0.55 | 0.546 | 0.545 | 0.545 | 0.545 | 0.545 | 0.545 | 0.545 | 0.545 | 0.545 | 0.545 | 0.55 | 0.546 | 0.545 | 0.546 | 0.545 | 0.545 | 0.545 | 0.549 | 0.545 | 0.545 | 0.545 | 0.549 | 0.545 | 0.549 |
| 9 | 0.61 | 0.61 | 0.609 | 0.614 | 0.61 | 0.604 | 0.61 | 0.61 | 0.61 | 0.61 | 0.609 | 0.61 | 0.61 | 0.61 | 0.615 | 0.61 | 0.609 | 0.61 | 0.61 | 0.61 | 0.609 | 0.614 | 0.61 | 0.609 | 0.61 | 0.614 | 0.61 | 0.611 |
| 10 | 0.652 | 0.653 | 0.652 | 0.658 | 0.653 | 0.652 | 0.652 | 0.652 | 0.652 | 0.652 | 0.652 | 0.652 | 0.652 | 0.652 | 0.658 | 0.653 | 0.652 | 0.653 | 0.652 | 0.652 | 0.652 | 0.657 | 0.652 | 0.652 | 0.652 | 0.657 | 0.652 | 0.656 |
| 11 | 0.793 | 0.794 | 0.793 | 0.805 | 0.794 | 0.794 | 0.794 | 0.794 | 0.794 | 0.794 | 0.793 | 0.793 | 0.793 | 0.793 | 0.8 | 0.794 | 0.793 | 0.794 | 0.793 | 0.793 | 0.793 | 0.8 | 0.793 | 0.793 | 0.793 | 0.8 | 0.793 | 0.798 |
| 12 | 0.951 | 0.951 | 0.951 | 0.955 | 0.951 | 0.951 | 0.951 | 0.95 | 0.951 | 0.951 | 0.951 | 0.951 | 0.95 | 0.951 | 0.959 | 0.951 | 0.95 | 0.951 | 0.951 | 0.951 | 0.951 | 0.959 | 0.951 | 0.95 | 0.951 | 0.959 | 0.95 | 0.956 |
| 13 | 1 | 1 | 1 | 1 | 1 | 1 | 1 | 1 | 1 | 1 | 1 | 1 | 1 | 1 | 1 | 1 | 1 | 1 | 1 | 1 | 1 | 1 | 1 | 1 | 1 | 1 | 1 | 1 |
| 14 | 1.093 | 1.092 | 1.093 | 1.101 | 1.093 | 1.093 | 1.093 | 1.092 | 1.093 | 1.093 | 1.092 | 1.092 | 1.092 | 1.092 | 1.102 | 1.093 | 1.092 | 1.093 | 1.093 | 1.092 | 1.093 | 1.102 | 1.092 | 1.092 | 1.092 | 1.102 | 1.092 | 1.099 |
| 15 | 1.14 | 1.14 | 1.14 | 1.149 | 1.14 | 1.14 | 1.14 | 1.14 | 1.14 | 1.14 | 1.14 | 1.14 | 1.14 | 1.14 | 1.15 | 1.14 | 1.14 | 1.14 | 1.14 | 1.14 | 1.14 | 1.15 | 1.14 | 1.14 | 1.14 | 1.15 | 1.14 | 1.149 |
| 16 | 1.172 | 1.201 | 1.172 | 1.181 | 1.173 | 1.187 | 1.187 | 1.186 | 1.172 | 1.187 | 1.172 | 1.186 | 1.172 | 1.172 | 1.182 | 1.188 | 1.184 | 1.172 | 1.187 | 1.172 | 1.172 | 1.182 | 1.172 | 1.172 | 1.172 | 1.182 | 1.172 | 1.18 |
| 17 | 1.35 | 1.349 | 1.349 | 1.361 | 1.35 | 1.349 | 1.349 | 1.348 | 1.35 | 1.349 | 1.349 | 1.348 | 1.349 | 1.349 | 1.361 | 1.349 | 1.346 | 1.35 | 1.348 | 1.349 | 1.349 | 1.362 | 1.348 | 1.35 | 1.35 | 1.362 | 1.348 | 1.359 |
| 18 | 1.379 | 1.379 | 1.379 | 1.39 | 1.379 | 1.379 | 1.379 | 1.379 | 1.379 | 1.379 | 1.379 | 1.379 | 1.379 | 1.379 | 1.389 | 1.379 | 1.379 | 1.379 | 1.379 | 1.379 | 1.379 | 1.391 | 1.378 | 1.379 | 1.379 | 1.391 | 1.378 | 1.389 |
| 19 | 1.433 | 1.433 | 1.432 | 1.444 | 1.433 | 1.433 | 1.433 | 1.433 | 1.433 | 1.433 | 1.432 | 1.433 | 1.433 | 1.433 | 1.445 | 1.433 | 1.432 | 1.433 | 1.433 | 1.433 | 1.433 | 1.446 | 1.433 | 1.432 | 1.432 | 1.445 | 1.432 | 1.442 |
| 20 | 1.571 | 1.572 | 1.571 | 1.583 | 1.571 | 1.571 | 1.572 | 1.571 | 1.572 | 1.571 | 1.571 | 1.572 | 1.572 | 1.572 | 1.583 | 1.572 | 1.571 | 1.571 | 1.572 | 1.571 | 1.571 | 1.585 | 1.571 | 1.571 | 1.571 | 1.586 | 1.57 | 1.581 |
| 21 | 1.606 | 1.601 | 1.606 | 1.618 | 1.606 | 1.601 | 1.606 | 1.606 | 1.607 | 1.601 | 1.606 | 1.602 | 1.606 | 1.606 | 1.618 | 1.606 | 1.606 | 1.601 | 1.602 | 1.601 | 1.601 | 1.615 | 1.606 | 1.606 | 1.606 | 1.615 | 1.605 | 1.617 |
| 22 | 1.664 | 1.664 | 1.664 | 1.677 | 1.664 | 1.664 | 1.665 | 1.664 | 1.665 | 1.664 | 1.664 | 1.665 | 1.665 | 1.665 | 1.677 | 1.665 | 1.664 | 1.664 | 1.665 | 1.664 | 1.664 | 1.68 | 1.664 | 1.664 | 1.664 | 1.679 | 1.664 | 1.675 |
| 23 | 1.708 | 1.708 | 1.707 | 1.719 | 1.708 | 1.708 | 1.708 | 1.707 | 1.708 | 1.708 | 1.707 | 1.708 | 1.708 | 1.708 | 1.712 | 1.708 | 1.706 | 1.708 | 1.708 | 1.707 | 1.708 | 1.724 | 1.707 | 1.706 | 1.706 | 1.723 | 1.706 | 1.718 |
| 24 | 1.732 | 1.732 | 1.731 | 1.744 | 1.732 | 1.731 | 1.732 | 1.731 | 1.732 | 1.731 | 1.731 | 1.732 | 1.732 | 1.732 | 1.745 | 1.732 | 1.731 | 1.731 | 1.732 | 1.732 | 1.732 | 1.747 | 1.731 | 1.731 | 1.731 | 1.746 | 1.731 | 1.742 |
| 25 | 1.828 | 1.827 | 1.828 | 1.841 | 1.827 | 1.827 | 1.827 | 1.827 | 1.827 | 1.827 | 1.827 | 1.828 | 1.827 | 1.827 | 1.841 | 1.827 | 1.826 | 1.826 | 1.828 | 1.827 | 1.827 | 1.843 | 1.827 | 1.826 | 1.826 | 1.843 | 1.826 | 1.838 |
| 26 | 1.977 | 1.977 | 1.977 | 1.991 | 1.977 | 1.977 | 1.978 | 1.977 | 1.978 | 1.977 | 1.977 | 1.978 | 1.978 | 1.978 | 1.992 | 1.977 | 1.977 | 1.977 | 1.978 | 1.977 | 1.977 | 1.995 | 1.977 | 1.976 | 1.976 | 1.994 | 1.976 | 1.989 |
| 27 | 2.015 | 2.014 | 2.015 | 2.029 | 2.015 | 2.014 | 2.015 | 2.014 | 2.015 | 2.014 | 2.015 | 2.015 | 2.015 | 2.015 | 2.029 | 2.014 | 2.014 | 2.014 | 2.015 | 2.015 | 2.015 | 2.032 | 2.014 | 2.014 | 2.014 | 2.032 | 2.014 | 2.027 |
| 28 | 2.089 | 2.089 | 2.089 | 2.104 | 2.089 | 2.089 | 2.089 | 2.089 | 2.089 | 2.088 | 2.089 | 2.09 | 2.09 | 2.09 | 2.104 | 2.089 | 2.089 | 2.088 | 2.09 | 2.089 | 2.089 | 2.107 | 2.089 | 2.088 | 2.088 | 2.107 | 2.088 | 2.101 |

Table S3 The peak area of common peaks of commercial rhubarb samples

| The ID of commercial rhubarb samples | | | | | | | | | | | | | | | | | | | | | | | | | | | | |
| --- | --- | --- | --- | --- | --- | --- | --- | --- | --- | --- | --- | --- | --- | --- | --- | --- | --- | --- | --- | --- | --- | --- | --- | --- | --- | --- | --- | --- |
| Peak No. | S1 | S2 | S3 | S4 | S5 | S6 | S7 | S8 | S9 | S10 | S11 | S12 | S13 | S14 | S15 | S16 | S17 | S18 | S19 | S20 | S21 | S22 | S23 | S24 | S25 | S26 | S27 | S28 |
| 1 | 0.083 | 0.049 | 0.171 | 1.288 | 0.081 | 0.091 | 0.075 | 0.024 | 0.018 | 0.035 | 0.086 | 0.018 | 0.151 | 0.045 | 0.11 | 0.031 | 0.015 | 0.013 | 0.031 | 0.022 | 0.058 | 0.077 | 0.025 | 0.125 | 0.03 | 0.427 | 0.03 | 0.119 |
| 2 | 1.625 | 0.222 | 0.752 | 2.189 | 1.854 | 0.389 | 0.063 | 0.083 | 0.476 | 0.189 | 1.086 | 0.158 | 0.373 | 0.161 | 1.506 | 0.097 | 0.114 | 0.186 | 0.097 | 0.14 | 0.482 | 2.931 | 0.245 | 6.496 | 1.472 | 0.489 | 0.209 | 0.712 |
| 3 | 0.648 | 0.49 | 3.254 | 32.934 | 0.66 | 1.99 | 1.285 | 0.471 | 0.344 | 0.808 | 1.092 | 0.528 | 0.65 | 0.869 | 0.499 | 0.551 | 0.473 | 0.365 | 0.623 | 0.555 | 1.394 | 0.563 | 0.613 | 0.524 | 0.238 | 24.072 | 0.51 | 5.705 |
| 4 | 0.072 | 0.044 | 0.161 | 0.525 | 0.14 | 0.1 | 0.047 | 0.022 | 0.03 | 0.039 | 0.209 | 0.044 | 0.04 | 0.043 | 0.247 | 0.032 | 0.022 | 0.022 | 0.03 | 0.031 | 0.034 | 0.45 | 0.027 | 0.523 | 0.051 | 0.303 | 0.027 | 0.488 |
| 5 | 0.402 | 0.249 | 0.869 | 1.933 | 0.554 | 1.199 | 0.568 | 0.235 | 0.343 | 0.43 | 0.582 | 0.364 | 0.39 | 0.463 | 4.814 | 0.228 | 0.328 | 0.387 | 0.324 | 0.359 | 0.805 | 8.571 | 0.396 | 2.791 | 0.425 | 7.319 | 0.38 | 2.909 |
| 6 | 0.016 | 0.033 | 0.079 | 0.745 | 0.041 | 0.171 | 0.06 | 0.032 | 0.036 | 0.05 | 0.061 | 0.033 | 0.058 | 0.057 | 0.946 | 0.029 | 0.028 | 0.035 | 0.037 | 0.046 | 0.06 | 1.881 | 0.039 | 0.383 | 0.052 | 1.202 | 0.051 | 3.694 |
| 7 | 0.018 | 0.024 | 0.099 | 0.994 | 0.089 | 0.079 | 0.071 | 0.024 | 0.016 | 0.031 | 0.065 | 0.038 | 0.037 | 0.027 | 1.773 | 0.012 | 0.013 | 0.014 | 0.014 | 0.017 | 0.036 | 0.55 | 0.022 | 0.23 | 0.03 | 1.061 | 0.039 | 0.255 |
| 8 | 0.014 | 0.071 | 0.056 | 5.146 | 0.083 | 0.188 | 0.14 | 0.066 | 0.059 | 0.097 | 0.069 | 0.075 | 0.142 | 0.059 | 4.2 | 0.045 | 0.053 | 0.051 | 0.057 | 0.063 | 0.181 | 5.968 | 0.09 | 0.347 | 0.03 | 3.411 | 0.129 | 1.277 |
| 9 | 0.101 | 0.05 | 0.168 | 1.33 | 0.289 | 0.058 | 0.063 | 0.053 | 0.05 | 0.05 | 0.219 | 0.042 | 0.061 | 0.041 | 0.253 | 0.035 | 0.052 | 0.04 | 0.039 | 0.045 | 0.058 | 0.436 | 0.037 | 0.361 | 0.156 | 0.28 | 0.047 | 0.092 |
| 10 | 0.116 | 0.215 | 0.366 | 2.873 | 0.264 | 0.602 | 0.356 | 0.19 | 0.191 | 0.362 | 0.291 | 0.269 | 0.383 | 0.261 | 7.341 | 0.149 | 0.211 | 0.204 | 0.206 | 0.264 | 0.705 | 7.26 | 0.338 | 1.158 | 0.144 | 5.055 | 0.315 | 1.322 |
| 11 | 0.27 | 0.031 | 1.031 | 17.958 | 0.97 | 0.034 | 0.014 | 0.014 | 0.107 | 0.022 | 0.655 | 0.011 | 0.121 | 0.023 | 2.325 | 0.009 | 0.01 | 0.018 | 0.012 | 0.02 | 0.012 | 2.277 | 0.018 | 2.084 | 0.267 | 1.562 | 0.035 | 0.212 |
| 12 | 0.073 | 0.063 | 0.642 | 14.406 | 0.267 | 0.096 | 0.047 | 0.029 | 0.125 | 0.055 | 0.447 | 0.031 | 0.101 | 0.034 | 6.573 | 0.036 | 0.032 | 0.037 | 0.027 | 0.035 | 0.031 | 9.8 | 0.026 | 0.906 | 0.038 | 8.874 | 0.05 | 1.979 |
| 13 | 1 | 1 | 1 | 1 | 1 | 1 | 1 | 1 | 1 | 1 | 1 | 1 | 1 | 1 | 1 | 1 | 1 | 1 | 1 | 1 | 1 | 1 | 1 | 1 | 1 | 1 | 1 | 1 |
| 14 | 0.081 | 0.062 | 0.719 | 1.666 | 0.262 | 0.125 | 0.064 | 0.026 | 0.099 | 0.017 | 0.449 | 0.018 | 0.065 | 0.015 | 3.822 | 0.007 | 0.009 | 0.016 | 0.01 | 0.012 | 0.01 | 3.669 | 0.014 | 0.582 | 0.048 | 8.329 | 0.054 | 1.848 |
| 15 | 0.122 | 0.503 | 0.389 | 1.188 | 0.063 | 1.053 | 0.532 | 0.29 | 0.077 | 0.381 | 0.151 | 0.382 | 0.576 | 0.298 | 0.165 | 0.317 | 0.253 | 0.152 | 0.301 | 0.281 | 0.745 | 0.148 | 0.275 | 0.144 | 0.094 | 0.676 | 0.206 | 1.364 |
| 16 | 0.107 | 0.121 | 0.356 | 59.288 | 0.27 | 0.144 | 0.088 | 0.046 | 0.079 | 0.046 | 0.194 | 0.05 | 0.121 | 0.022 | 2.057 | 0.045 | 0.041 | 0.018 | 0.071 | 0.016 | 0.028 | 1.63 | 0.025 | 0.899 | 0.132 | 1.235 | 0.024 | 6.724 |
| 17 | 0.235 | 0.108 | 1.284 | 13.86 | 0.535 | 0.237 | 0.124 | 0.073 | 0.198 | 0.087 | 0.509 | 0.05 | 0.309 | 0.108 | 1.724 | 0.082 | 0.039 | 0.042 | 0.067 | 0.081 | 0.084 | 3.348 | 0.075 | 1.32 | 0.235 | 3.672 | 0.075 | 5.214 |
| 18 | 0.448 | 0.145 | 2.319 | 26.149 | 0.775 | 0.202 | 0.121 | 0.08 | 0.238 | 0.076 | 0.771 | 0.054 | 0.438 | 0.202 | 0.933 | 0.105 | 0.068 | 0.082 | 0.076 | 0.167 | 0.136 | 2.172 | 0.11 | 2.539 | 0.533 | 1.908 | 0.108 | 10.583 |
| 19 | 0.187 | 0.221 | 0.87 | 1.126 | 0.465 | 0.168 | 0.095 | 0.151 | 0.354 | 0.179 | 0.411 | 0.085 | 0.567 | 0.183 | 3.599 | 0.152 | 0.129 | 0.101 | 0.113 | 0.166 | 0.251 | 3.283 | 0.142 | 1.323 | 0.219 | 5.326 | 0.114 | 10.602 |
| 20 | 0.301 | 0.071 | 0.536 | 4.238 | 0.435 | 0.082 | 0.056 | 0.032 | 0.075 | 0.073 | 0.383 | 0.025 | 0.378 | 0.145 | 0.094 | 0.032 | 0.03 | 0.026 | 0.039 | 0.083 | 0.077 | 0.987 | 0.08 | 2.988 | 0.629 | 0.605 | 0.093 | 0.309 |
| 21 | 0.186 | 0.106 | 0.746 | 14.085 | 0.433 | 0.407 | 0.078 | 0.039 | 0.181 | 0.106 | 0.295 | 0.042 | 0.314 | 0.093 | 1.344 | 0.06 | 0.018 | 0.047 | 0.083 | 0.119 | 0.096 | 8.322 | 0.058 | 1.042 | 0.201 | 9.342 | 0.052 | 12.781 |
| 22 | 0.11 | 0.273 | 1.697 | 6.905 | 0.475 | 0.548 | 0.292 | 0.208 | 0.078 | 0.289 | 0.587 | 0.161 | 0.315 | 0.193 | 0.764 | 0.164 | 0.172 | 0.118 | 0.26 | 0.193 | 0.195 | 0.389 | 0.144 | 0.563 | 0.108 | 4.645 | 0.176 | 1.357 |
| 23 | 0.262 | 0.215 | 2.003 | 0.117 | 0.123 | 0.704 | 0.307 | 0.167 | 0.087 | 0.217 | 0.498 | 0.145 | 0.439 | 0.302 | 0.027 | 0.169 | 0.12 | 0.075 | 0.245 | 0.187 | 0.11 | 0.035 | 0.106 | 0.161 | 0.102 | 0.193 | 0.105 | 0.168 |
| 24 | 0.15 | 0.046 | 0.327 | 2.791 | 0.317 | 0.107 | 0.044 | 0.021 | 0.093 | 0.048 | 0.239 | 0.023 | 0.379 | 0.09 | 0.87 | 0.04 | 0.015 | 0.02 | 0.024 | 0.043 | 0.06 | 1.434 | 0.055 | 1.851 | 0.371 | 0.942 | 0.065 | 0.756 |
| 25 | 0.07 | 0.394 | 0.903 | 0.909 | 0.1 | 1.351 | 0.514 | 0.371 | 0.072 | 0.501 | 0.245 | 0.367 | 0.277 | 0.266 | 1.362 | 0.253 | 0.311 | 0.237 | 0.412 | 0.309 | 0.388 | 1.302 | 0.279 | 0.079 | 0.014 | 13.63 | 0.236 | 3.36 |
| 26 | 0.093 | 0.5 | 1.13 | 12.583 | 0.288 | 1.49 | 0.593 | 0.628 | 0.197 | 0.635 | 0.394 | 0.39 | 0.696 | 0.49 | 1.095 | 0.411 | 0.449 | 0.223 | 0.583 | 0.476 | 0.787 | 0.484 | 0.413 | 0.349 | 0.092 | 9.625 | 0.311 | 11.548 |
| 27 | 0.479 | 0.73 | 4.404 | 24.709 | 1.248 | 1.726 | 1.028 | 0.64 | 0.259 | 0.905 | 1.539 | 0.51 | 1.598 | 0.903 | 0.99 | 0.392 | 0.556 | 0.397 | 0.627 | 0.464 | 0.979 | 1.33 | 0.584 | 2.27 | 0.624 | 9.262 | 0.691 | 10.152 |
| 28 | 0.15 | 0.258 | 1.515 | 7.328 | 0.458 | 0.5 | 0.402 | 0.219 | 0.095 | 0.219 | 0.499 | 0.196 | 0.595 | 0.302 | 0.543 | 0.084 | 0.166 | 0.126 | 0.242 | 0.117 | 0.279 | 0.467 | 0.202 | 0.748 | 0.193 | 3.992 | 0.19 | 6.054 |

Table S4 The value of four DL properties of the 28 commercial rhubarb samples

| Sample | Species | I0 | Tau | Beta | T |
| --- | --- | --- | --- | --- | --- |
| S1 | *Rheum officinale* Bail. | 6558.733 | 1.005429 | 3.391366 | 0.344753 |
| S2 | *Rheum palmatum* L. | 5422.063 | 0.91239 | 3.130911 | 0.343302 |
| S3 | *Rheum tanguticum* Maxim. ex Balf. | 3026.182 | 1.16484 | 3.421982 | 0.395033 |
| S4 | *Rheum tanguticum* Maxim. ex Balf. | 523.1962 | 0.609093 | 2.598986 | 0.284916 |
| S5 | *Rheum officinale* Bail. | 4613.278 | 1.092787 | 3.332122 | 0.382316 |
| S6 | *Rheum palmatum* L. | 1206.206 | 0.877658 | 3.113392 | 0.331877 |
| S7 | *Rheum palmatum* L. | 7067.671 | 1.149836 | 3.532305 | 0.376181 |
| S8 | *Rheum officinale* Bail. | 10073.97 | 1.050448 | 3.432466 | 0.355095 |
| S9 | *Rheum tanguticum* Maxim. ex Balf. | 11589.88 | 1.270847 | 3.624769 | 0.403382 |
| S10 | *Rheum palmatum* L. | 26460.39 | 1.414684 | 3.94738 | 0.407752 |
| S11 | *Rheum officinale* Bail. | 5393.16 | 1.119268 | 3.3479 | 0.389606 |
| S12 | *Rheum tanguticum* Maxim. ex Balf. | 7164.697 | 1.176375 | 3.701287 | 0.364828 |
| S13 | *Rheum officinale* Bail. | 5525.424 | 0.783216 | 2.837452 | 0.330837 |
| S14 | *Rheum officinale* Bail. | 10272.62 | 1.198789 | 3.6624 | 0.376361 |
| S15 | *Rheum tanguticum* Maxim. ex Balf. | 1161.514 | 0.564555 | 2.552929 | 0.270215 |
| S16 | *Rheum officinale* Bail. | 12223.85 | 1.073691 | 3.471519 | 0.358354 |
| S17 | *Rheum officinale* Bail. | 17678.87 | 1.231559 | 3.785623 | 0.37216 |
| S18 | *Rheum palmatum* L. | 12055.68 | 1.173514 | 3.728934 | 0.360641 |
| S19 | *Rheum palmatum* L. | 15807.01 | 1.200933 | 3.577541 | 0.387005 |
| S20 | *Rheum tanguticum* Maxim. ex Balf. | 15470.07 | 1.265917 | 3.77603 | 0.3838 |
| S21 | *Rheum officinale* Bail. | 14068.25 | 1.193275 | 3.653003 | 0.375678 |
| S22 | *Rheum tanguticum* Maxim. ex Balf. | 553.5528 | 0.911554 | 3.153273 | 0.340082 |
| S23 | *Rheum palmatum* L. | 6834.318 | 0.998634 | 3.310992 | 0.352078 |
| S24 | *Rheum officinale* Bail. | 16847.89 | 1.702377 | 4.104661 | 0.469464 |
| S25 | *Rheum officinale* Bail. | 8120.601 | 1.455701 | 3.885696 | 0.427147 |
| S26 | *Rheum officinale* Bail. | 104.5544 | 0.208927 | 1.324945 | 0.231747 |
| S27 | *Rheum officinale* Bail. | 26377.63 | 1.5035 | 4.08575 | 0.416884 |
| S28 | *Rheum officinale* Bail. | 170.7197 | 0.258647 | 1.664263 | 0.200549 |

Table S5 The correlation coefficient between DL properties and chemical compounds

| DL | Compound | Correlation coefficient |
| --- | --- | --- |
| I0 | Sennoside A | -0.72697 |
| I0 | Chrysophanol-1-O-beta-glu | -0.63638 |
| I0 | Physcion-8-O-beta-glu | -0.56865 |
| I0 | Rhein-8-O-beta-glu | -0.52888 |
| Beta | Sennoside A | -0.52552 |
| I0 | Emodin-1-O-beta-glu | -0.50986 |
| Beta | Chrysophanol-1-O-beta-glu | -0.50007 |
| Beta | Physcion-8-O-beta-glu | -0.4616 |
| Beta | Emodin-1-O-beta-glu | -0.43072 |
| I0 | Aloe-emodin-8-O-beta-glu | -0.42528 |
| Tau | Sennoside A | -0.4248 |
| Beta | Rhein-8-O-beta-glu | -0.39009 |
| Tau | Chrysophanol-1-O-beta-glu | -0.38566 |
| I0 | Chrysophanol-8-O-beta-glu | -0.34921 |
| Tau | Rhein-8-O-beta-glu | -0.34492 |
| Tau | Physcion-8-O-beta-glu | -0.33539 |
| Tau | Emodin-1-O-beta-glu | -0.32913 |

Table S6 The value of DL properties and chemical components of the 118 wild rhubarb samples

| No | Species | I0 | Tau | Beta | T | Total anthraquinone glycosides (mg/g) | Total free anthraquinones (mg/g) | Total phenolics (mg/g) |
| --- | --- | --- | --- | --- | --- | --- | --- | --- |
| 1 | *Rheum palmatum* L. | 277.9327 | 0.58952 | 2.724406 | 0.255702 | 42.99083509 | 10.8060292 | 1.52197219 |
| 2 | *Rheum palmatum* L. | 1037.191 | 1.060098 | 3.425491 | 0.357535 | 50.03555886 | 10.98538674 | 4.01463145 |
| 3 | *Rheum palmatum* L. | 185.5201 | 0.213595 | 1.387046 | 0.224821 | 53.19551337 | 16.82930861 | 2.03944182 |
| 4 | *Rheum palmatum* L. | 1391.66 | 0.742539 | 2.664823 | 0.338079 | 36.624 | 13.203 | 3.08 |
| 5 | *Rheum palmatum* L. | 6464.339 | 0.901391 | 3.012404 | 0.354915 | 33.614 | 9.583 | 0.821 |
| 6 | *Rheum palmatum* L. | 6074.701 | 0.816984 | 2.893324 | 0.337257 | 34.792 | 11.153 | 3.53 |
| 7 | *Rheum palmatum* L. | 3710.741 | 0.865923 | 2.955127 | 0.348559 | 29.98 | 16.32 | 0.66 |
| 8 | *Rheum palmatum* L. | 1708.365 | 0.796788 | 3.200331 | 0.292031 | 20.595 | 3.827 | 1.06 |
| 9 | *Rheum palmatum* L. | 1571.21 | 0.608453 | 2.615506 | 0.283192 | 25.622 | 3.957 | 1.38 |
| 10 | *Rheum palmatum* L. | 2748.202 | 0.649252 | 2.945217 | 0.261802 | 28.983 | 2.151 | 1.163 |
| 11 | *Rheum palmatum* L. | 7544.781 | 0.728236 | 3.00503 | 0.28742 | 26.262 | 2.314 | 1.37 |
| 12 | *Rheum palmatum* L. | 3042.059 | 0.59235 | 2.682986 | 0.267505 | 20.61 | 3.7051 | 0.711 |
| 13 | *Rheum palmatum* L. | 6816.228 | 0.828611 | 3.178587 | 0.306227 | 61.255 | 4.261 | 0.903 |
| 14 | *Rheum palmatum* L. | 6116.704 | 0.849194 | 2.983136 | 0.338157 | 65.461 | 5.489 | 0.954 |
| 15 | *Rheum palmatum* L. | 5210.83 | 0.750791 | 2.960553 | 0.301524 | 45.755 | 1.518 | 0.793 |
| 16 | *Rheum palmatum* L. | 7751.116 | 0.767875 | 3.053604 | 0.29725 | 32.078 | 1.8 | 1.233 |
| 17 | *Rheum palmatum* L. | 3918.199 | 0.849496 | 3.169278 | 0.315139 | 29.294 | 3.376 | 0.813 |
| 18 | *Rheum palmatum* L. | 588.0426 | 0.50362 | 2.701407 | 0.222408 | 62.50197989 | 5.68884139 | 2.56348172 |
| 19 | *Rheum palmatum* L. | 319.654 | 0.249331 | 1.748168 | 0.189548 | 47.73947987 | 8.13422261 | 1.827836 |
| 20 | *Rheum palmatum* L. | 302.5605 | 0.217654 | 1.791726 | 0.159813 | 59.9444598 | 12.12433079 | 2.60625902 |
| 21 | *Rheum palmatum* L. | 320.6172 | 0.204808 | 1.685391 | 0.157613 | 43.79892747 | 7.84355377 | 3.321779 |
| 22 | *Rheum palmatum* L. | 274.3853 | 0.189616 | 1.626403 | 0.160285 | 30.18572682 | 6.0225706 | 2.39963698 |
| 23 | *Rheum palmatum* L. | 1245.044 | 1.262861 | 3.717281 | 0.388201 | 69.35972703 | 7.04352861 | 5.00034783 |
| 24 | *Rheum palmatum* L. | 130.4865 | 0.10494 | 1.022533 | 0.171494 | 65.50040358 | 7.21974849 | 2.18922377 |
| 25 | *Rheum palmatum* L. | 133.3223 | 0.139153 | 1.07981 | 0.210868 | 63.09293729 | 9.21189731 | 2.15220644 |
| 26 | *Rheum palmatum* L. | 163.5306 | 0.130127 | 1.166736 | 0.176662 | 37.04990481 | 9.17202013 | 4.16472874 |
| 27 | *Rheum palmatum* L. | 240.6403 | 0.316036 | 1.73909 | 0.244688 | 59.96303192 | 8.25988104 | 4.7389117 |
| 28 | *Rheum palmatum* L. | 317.6866 | 0.265073 | 1.755764 | 0.202928 | 48.29183984 | 6.043103298 | 5.037121597 |
| 29 | *Rheum palmatum* L. | 530.5119 | 0.402977 | 2.198634 | 0.22978 | 66.32475988 | 7.883501952 | 3.413314784 |
| 30 | *Rheum palmatum* L. | 742.2617 | 0.385592 | 2.19203 | 0.220276 | 55.27518744 | 7.706665473 | 3.862632418 |
| 31 | *Rheum palmatum* L. | 655.5809 | 0.467291 | 2.432291 | 0.236932 | 53.43211242 | 8.453154021 | 3.208395393 |
| 32 | *Rheum palmatum* L. | 619.8844 | 0.407817 | 2.252172 | 0.224964 | 39.47242092 | 4.942311169 | 4.650301029 |
| 33 | *Rheum palmatum* L. | 692.1403 | 0.527051 | 2.468733 | 0.261931 | 117.169 | 9.157 | 4.06 |
| 34 | *Rheum palmatum* L. | 463.6108 | 0.639799 | 2.644701 | 0.293945 | 60.116 | 8.698 | 2.43 |
| 35 | *Rheum palmatum* L. | 202.5161 | 0.121377 | 1.244901 | 0.144428 | 42.99083509 | 10.8060292 | 1.52197219 |
| 36 | *Rheum palmatum* L. | 127.6775 | 0.096242 | 1.048587 | 0.150451 | 50.03555886 | 10.98538674 | 4.01463145 |
| 37 | *Rheum palmatum* L. | 197.5558 | 0.125059 | 1.245918 | 0.143339 | 75.52064307 | 11.19028427 | 11.242052 |
| 38 | *Rheum palmatum* L. | 264.324 | 0.186254 | 1.584047 | 0.150816 | 73.14344494 | 11.89955306 | 4.063031898 |
| 39 | *Rheum palmatum* L. | 327.0034 | 0.2569 | 1.770873 | 0.193351 | 73.54687852 | 9.029100716 | 6.881060356 |
| 40 | *Rheum palmatum* L. | 311.1197 | 0.32953 | 1.771587 | 0.246453 | 58.56808609 | 9.906019709 | 7.443760064 |
| 41 | *Rheum palmatum* L. | 152.3323 | 0.031185 | 0.805097 | 0.076803 | 48.58543942 | 12.56499741 | 3.646801118 |
| 42 | *Rheum palmatum* L. | 446.9764 | 0.569622 | 2.420787 | 0.290479 | 35.58931257 | 10.59746932 | 5.094190651 |
| 43 | *Rheum palmatum* L. | 303.4363 | 0.315564 | 1.85294 | 0.214422 | 33.15388236 | 8.912132951 | 4.657613368 |
| 44 | *Rheum palmatum* L. | 117.3156 | 0.105439 | 1.030451 | 0.17212 | 50.5455278 | 11.69801944 | 6.46141161 |
| 45 | *Rheum palmatum* L. | 274.5155 | 0.416701 | 2.091668 | 0.253559 | 47.05573714 | 6.83173643 | 5.52462179 |
| 46 | *Rheum palmatum* L. | 419.464 | 0.534285 | 2.503876 | 0.25873 | 40.79164968 | 10.73692376 | 3.09616126 |
| 47 | *Rheum palmatum* L. | 414.414 | 0.102359 | 1.4239 | 0.104165 | 45.5467985 | 6.71217292 | 3.74979482 |
| 48 | *Rheum palmatum* L. | 3001.568 | 0.628357 | 2.874473 | 0.260416 | 59.846572 | 7.283845022 | 6.136821885 |
| 49 | *Rheum palmatum* L. | 4449.227 | 0.775017 | 3.00727 | 0.305671 | 75.26368475 | 12.08445431 | 4.10224898 |
| 50 | *Rheum palmatum* L. | 5249.82 | 0.827344 | 2.948765 | 0.333907 | 31.83700846 | 7.371097221 | 4.029701526 |
| 51 | *Rheum tanguticum* Maxim. ex Balf. | 2119.754 | 0.826861 | 3.109397 | 0.313091 | 54.06327683 | 7.263486346 | 3.213747155 |
| 52 | *Rheum tanguticum* Maxim. ex Balf. | 1194.804 | 0.634294 | 2.706723 | 0.283239 | 43.51650331 | 8.516259717 | 2.633886004 |
| 53 | *Rheum tanguticum* Maxim. ex Balf. | 172.3091 | 0.165817 | 1.307375 | 0.189768 | 54.03830813 | 6.802194701 | 5.968678387 |
| 54 | *Rheum tanguticum* Maxim. ex Balf. | 1344.26 | 0.781266 | 3.043871 | 0.302894 | 62.13466195 | 5.651561691 | 3.098204843 |
| 55 | *Rheum tanguticum* Maxim. ex Balf. | 245.0448 | 0.165899 | 1.408968 | 0.170895 | 55.16578078 | 6.805804955 | 3.573408907 |
| 56 | *Rheum tanguticum* Maxim. ex Balf. | 218.2895 | 0.836996 | 2.917559 | 0.34111 | 33.75320722 | 18.90905925 | 2.668327913 |
| 57 | *Rheum tanguticum* Maxim. ex Balf. | 1034.518 | 1.072677 | 3.470326 | 0.357304 | 45.49816298 | 8.362157515 | 0.792371633 |
| 58 | *Rheum tanguticum* Maxim. ex Balf. | 1197.156 | 0.69493 | 2.852535 | 0.291676 | 54.21548628 | 5.517974576 | 2.287608706 |
| 59 | *Rheum tanguticum* Maxim. ex Balf. | 512.2628 | 0.742188 | 2.837823 | 0.312 | 53.78932031 | 6.766406506 | 2.507902507 |
| 60 | *Rheum tanguticum* Maxim. ex Balf. | 2686.046 | 1.077529 | 3.465056 | 0.36046 | 35.48084713 | 6.33388521 | 0.756212091 |
| 61 | *Rheum tanguticum* Maxim. ex Balf. | 6382.822 | 0.780933 | 3.08874 | 0.298264 | 69.23210971 | 10.02019818 | 1.027714517 |
| 62 | *Rheum tanguticum* Maxim. ex Balf. | 592.6229 | 0.489937 | 2.280453 | 0.266422 | 40.71027053 | 19.30805021 | 0.751646117 |
| 63 | *Rheum tanguticum* Maxim. ex Balf. | 2189.086 | 0.667634 | 2.733452 | 0.294766 | 65.67985188 | 4.077352059 | 1.541950236 |
| 64 | *Rheum tanguticum* Maxim. ex Balf. | 2302.078 | 0.723178 | 2.920026 | 0.29521 | 73.43960654 | 14.02453847 | 2.616859448 |
| 65 | *Rheum tanguticum* Maxim. ex Balf. | 19188.15 | 1.363675 | 3.800559 | 0.410434 | 40.11813028 | 10.55684434 | 0.643087932 |
| 66 | *Rheum tanguticum* Maxim. ex Balf. | 16232.67 | 1.292214 | 3.87482 | 0.380395 | 31.34657225 | 9.257358068 | 1.914438639 |
| 67 | *Rheum tanguticum* Maxim. ex Balf. | 137.7601 | 0.089647 | 1.079129 | 0.12599 | 102.0996644 | 11.54594138 | 3.714553171 |
| 68 | *Rheum tanguticum* Maxim. ex Balf. | 244.9244 | 0.409565 | 2.359573 | 0.208215 | 90.32640063 | 13.46114987 | 2.964094148 |
| 69 | *Rheum tanguticum* Maxim. ex Balf. | 211.3089 | 0.440249 | 2.178771 | 0.248327 | 74.765827 | 14.20652608 | 1.979874173 |
| 70 | *Rheum tanguticum* Maxim. ex Balf. | 278.025 | 0.442147 | 2.250561 | 0.246717 | 87.29809676 | 10.78263757 | 3.515037176 |
| 71 | *Rheum tanguticum* Maxim. ex Balf. | 311.4923 | 0.411675 | 2.080581 | 0.253935 | 62.8151527 | 20.59083793 | 5.865909292 |
| 72 | *Rheum tanguticum* Maxim. ex Balf. | 429.9244 | 0.439203 | 2.217078 | 0.250381 | 40.70605525 | 15.38501045 | 8.036849702 |
| 73 | *Rheum tanguticum* Maxim. ex Balf. | 213.0951 | 0.230354 | 1.486199 | 0.217506 | 44.69231481 | 12.31550482 | 4.80361449 |
| 74 | *Rheum tanguticum* Maxim. ex Balf. | 232.0665 | 0.233522 | 1.526464 | 0.216052 | 37.66616952 | 17.33319304 | 3.237667293 |
| 75 | *Rheum tanguticum* Maxim. ex Balf. | 173.1548 | 0.141029 | 1.223899 | 0.176281 | 45.97768814 | 13.94957728 | 3.359231364 |
| 76 | *Rheum tanguticum* Maxim. ex Balf. | 158.4863 | 0.152887 | 1.224511 | 0.189728 | 66.51096312 | 21.96501664 | 3.85957396 |
| 77 | *Rheum tanguticum* Maxim. ex Balf. | 1425.452 | 0.614311 | 2.569281 | 0.291719 | 67.24290261 | 26.10398194 | 13.79925569 |
| 78 | *Rheum tanguticum* Maxim. ex Balf. | 171.0199 | 0.056764 | 0.900699 | 0.099827 | 74.78230981 | 11.99508015 | 4.766306976 |
| 79 | *Rheum tanguticum* Maxim. ex Balf. | 408.7244 | 0.278214 | 1.792991 | 0.206816 | 58.22132344 | 4.111155105 | 3.179211681 |
| 80 | *Rheum tanguticum* Maxim. ex Balf. | 318.1147 | 0.331433 | 1.918667 | 0.223724 | 43.26457963 | 6.982830553 | 4.941665859 |
| 81 | *Rheum tanguticum* Maxim. ex Balf. | 170.9311 | 0.340586 | 1.842733 | 0.245426 | 56.83320165 | 2.843905344 | 2.122512197 |
| 82 | *Rheum tanguticum* Maxim. ex Balf. | 255.201 | 0.328475 | 1.811805 | 0.23872 | 59.13630585 | 8.339350661 | 2.350153754 |
| 83 | *Rheum tanguticum* Maxim. ex Balf. | 619.0801 | 0.540016 | 2.456288 | 0.27133 | 45.77367456 | 4.84397516 | 3.024464654 |
| 84 | *Rheum tanguticum* Maxim. ex Balf. | 265.8944 | 0.221993 | 1.612807 | 0.18781 | 55.13583321 | 6.044777203 | 3.919794085 |
| 85 | *Rheum tanguticum* Maxim. ex Balf. | 210.3028 | 0.132474 | 1.273988 | 0.157041 | 59.60337845 | 4.691047573 | 4.059207656 |
| 86 | *Rheum tanguticum* Maxim. ex Balf. | 1499.903 | 0.585964 | 2.605772 | 0.271931 | 39.8558255 | 3.638994453 | 1.732560455 |
| 87 | *Rheum tanguticum* Maxim. ex Balf. | 305.1933 | 0.400472 | 2.077544 | 0.247561 | 46.37977785 | 10.29882726 | 0.497 |
| 88 | *Rheum tanguticum* Maxim. ex Balf. | 298.3551 | 0.351282 | 1.925939 | 0.238626 | 47.68459523 | 6.40603934 | 4.451609282 |
| 89 | *Rheum tanguticum* Maxim. ex Balf. | 176.6188 | 0.195366 | 1.448963 | 0.189849 | 41.01511371 | 6.229640513 | 1.853228441 |
| 90 | *Rheum tanguticum* Maxim. ex Balf. | 216.3943 | 0.237666 | 1.650537 | 0.193778 | 53.55895613 | 6.409354986 | 2.175191207 |
| 91 | *Rheum tanguticum* Maxim. ex Balf. | 570.4714 | 0.618476 | 2.776359 | 0.266522 | 49.59841908 | 8.547890851 | 3.377372273 |
| 92 | *Rheum tanguticum* Maxim. ex Balf. | 630.57 | 0.446159 | 2.355079 | 0.23563 | 59.18966213 | 9.075739282 | 1.777395552 |
| 93 | *Rheum tanguticum* Maxim. ex Balf. | 217.2493 | 0.459008 | 2.306338 | 0.244973 | 85.11513632 | 10.60374275 | 3.423348522 |
| 94 | *Rheum tanguticum* Maxim. ex Balf. | 1333.396 | 0.668748 | 2.852776 | 0.280445 | 40.62711061 | 11.00200402 | 2.855613946 |
| 95 | *Rheum tanguticum* Maxim. ex Balf. | 370.5165 | 0.482558 | 2.260611 | 0.256973 | 43.14708146 | 13.94031761 | 2.280057741 |
| 96 | *Rheum tanguticum* Maxim. ex Balf. | 1846.943 | 0.508299 | 2.438621 | 0.25747 | 49.52379948 | 9.095317901 | 2.192132198 |
| 97 | *Rheum tanguticum* Maxim. ex Balf. | 1141.461 | 0.564148 | 2.598609 | 0.264015 | 47.14827519 | 5.05381412 | 1.6165416 |
| 98 | *Rheum tanguticum* Maxim. ex Balf. | 472.119 | 0.415181 | 2.092006 | 0.254051 | 84.36139021 | 13.15202825 | 1.400910155 |
| 99 | *Rheum tanguticum* Maxim. ex Balf. | 197.1809 | 0.197553 | 1.409334 | 0.199057 | 51.92620639 | 10.45337728 | 6.498516168 |
| 100 | *Rheum tanguticum* Maxim. ex Balf. | 184.119 | 0.243082 | 1.510415 | 0.220476 | 49.31716313 | 3.572497806 | 12.11443924 |
| 101 | *Rheum tanguticum* Maxim. ex Balf. | 268.2394 | 0.325077 | 1.81886 | 0.235971 | 48.83856978 | 2.851214289 | 7.232009662 |
| 102 | *Rheum tanguticum* Maxim. ex Balf. | 383.9261 | 0.410787 | 2.089461 | 0.250394 | 81.02792217 | 9.533201873 | 10.07928434 |
| 103 | *Rheum tanguticum* Maxim. ex Balf. | 324.1238 | 0.260316 | 1.689702 | 0.204962 | 60.5341993 | 6.45688866 | 9.848686315 |
| 104 | *Rheum tanguticum* Maxim. ex Balf. | 1323.663 | 0.725619 | 3.088709 | 0.275919 | 34.45282436 | 6.960896097 | 4.270166269 |
| 105 | *Rheum tanguticum* Maxim. ex Balf. | 3890.013 | 0.584464 | 2.704565 | 0.261351 | 29.19215823 | 10.59003577 | 6.237482191 |
| 106 | *Rheum tanguticum* Maxim. ex Balf. | 201.2194 | 0.177851 | 1.433489 | 0.178977 | 29.11616672 | 7.523739302 | 3.04048697 |
| 107 | *Rheum tanguticum* Maxim. ex Balf. | 2913.455 | 0.585242 | 2.795901 | 0.251503 | 48.01606886 | 11.75633846 | 1.83927152 |
| 108 | *Rheum tanguticum* Maxim. ex Balf. | 2827.664 | 0.958921 | 3.583589 | 0.306512 | 67.33260447 | 6.345890795 | 0.817235304 |
| 109 | *Rheum tanguticum* Maxim. ex Balf. | 5478.182 | 1.253235 | 3.785509 | 0.378613 | 45.75484846 | 5.827544422 | 0.791056857 |
| 110 | *Rheum tanguticum* Maxim. ex Balf. | 137.8122 | 0.147169 | 1.250153 | 0.174458 | 69.31290186 | 4.29576229 | 1.838092292 |
| 111 | *Rheum tanguticum* Maxim. ex Balf. | 142.8997 | 0.294848 | 1.60055 | 0.253942 | 62.20811975 | 6.38310483 | 6.745904218 |
| 112 | *Rheum tanguticum* Maxim. ex Balf. | 151.2873 | 0.324665 | 1.685703 | 0.249142 | 36.76870025 | 5.153356612 | 3.23671623 |
| 113 | *Rheum tanguticum* Maxim. ex Balf. | 227.8894 | 0.364959 | 2.029185 | 0.222786 | 52.4049727 | 4.591088354 | 5.413633916 |
| 114 | *Rheum palmatum* L. | 214.5446 | 0.311838 | 1.796124 | 0.228412 | 61.24590587 | 4.26368921 | 0.9254276 |
| 115 | *Rheum palmatum* L. | 208.4312 | 0.362525 | 1.856031 | 0.254186 | 65.46631708 | 5.48662521 | 0.97313884 |
| 116 | *Rheum palmatum* L. | 127.2576 | 0.223729 | 1.423927 | 0.22359 | 45.1797326 | 1.523299975 | 0.799264136 |
| 117 | *Rheum palmatum* L. | 242.4654 | 0.341105 | 1.908002 | 0.234447 | 31.62816438 | 1.809642423 | 1.226635914 |
| 118 | *Rheum palmatum* L. | 262.6975 | 0.670788 | 2.486056 | 0.329993 | 37.66300111 | 3.854979276 | 0.780785635 |
